# Cancer cell CCL5 mediates bone marrow independent angiogenesis in breast cancer

**DOI:** 10.18632/oncotarget.13387

**Published:** 2016-11-16

**Authors:** Michael John Sax, Christin Gasch, Vineel Rag Athota, Ruth Freeman, Parisa Rasighaemi, David Elton Westcott, Christopher John Day, Iva Nikolic, Benjamin Elsworth, Ming Wei, Kelly Rogers, Alexander Swarbrick, Vivek Mittal, Normand Pouliot, Albert Sleiman Mellick

**Affiliations:** ^1^ School of Medical Science, Griffith University, Gold Coast, QLD, Australia; ^2^ School of Medicine, Deakin University, Waurn Ponds, Victoria, Australia; ^3^ Glycomics Institute, Griffith University, Gold Coast, QLD, Australia; ^4^ Kinghorn Cancer Centre & Cancer Research Division, Garvan Institute of Medical Research, Darlinghurst, NSW, Australia; ^5^ St Vincent's Clinical School, Faculty of Medicine, University of New South Wales, Kensington NSW, Australia; ^6^ Centre for Dynamic Imaging, Walter and Eliza Hall Institute for Medical Research, Parkville Victoria, Australia; ^7^ Cardiothoracic Surgery and Neuberger Berman Lung Cancer Centre, Weill Cornell Medical College, New York, NY, USA; ^8^ Matrix Microenvironment & Metastasis Laboratory, Olivia Newton-John Cancer Research Institute, Heidelberg, Victoria, Australia; ^9^ School of Cancer Medicine, La Trobe University, Heidelberg, Victoria, Australia; ^10^ Faculty of Medicine, University of New South Wales, NSW, Australia; ^11^ School of Medicine, Western Sydney University, Campbelltown NSW, Australia; ^12^ Translational Oncology Unit, Ingham Institute for Applied Medical Research, Liverpool NSW, Australia

**Keywords:** angiogenesis, shRNAi, breast cancer, CCL5, CCR5

## Abstract

It has recently been suggested that the chemokine receptor (CCR5) is required for bone marrow (BM) derived endothelial progenitor cell (EPC) mediated angiogenesis. Here we show that suppression of either cancer cell produced CCL5, or host CCR5 leads to distinctive vascular and tumor growth defects in breast cancer. Surprisingly, CCR5 restoration in the BM alone was not sufficient to rescue the wild type phenotype, suggesting that impaired tumor growth associated with inhibiting CCL5/CCR5 is not due to defects in EPC biology. Instead, to promote angiogenesis cancer cell CCL5 may signal directly to endothelium in the tumor-stroma. In support of this hypothesis, we have also shown: (i) that endothelial cell CCR5 levels increases in response to tumor-conditioned media; (ii) that the amount of CCR5^+^ tumor vasculature correlates with invasive grade; and (iii) that inhibition of CCL5/CCR5 signaling impairs endothelial cell migration, associated with a decrease in activation of mTOR/AKT pathway members. Finally, we show that treatment with CCR5 antagonist results in less vasculature, impaired tumor growth, reduced metastases and improved survival. Taken as a whole, this work demonstrates that directly inhibiting CCR5 expressing vasculature constitutes a novel strategy for inhibiting angiogenesis and blocking metastatic progression in breast cancer.

## INTRODUCTION

For solid tumors to grow and spread they produce pro-angiogenic growth factors, such as vascular endothelial growth factor (VEGF) that promote the activation of surrounding host endothelial cells, as well as the recruitment of bone marrow (BM) derived endothelial progenitor cells (EPCs) [1-6]. Blocking VEGF/VEGF receptor 2 (VEGFR2) signaling using anti-angiogenic agents such as bevacizumab leads to defects in EPC biology, as well as impaired tumor growth and spread [7, 8]. However, many tumor types including those of the breast are non-responsive to treatment, while others develop resistance. Thus, the clinical benefit of bevacizumab remains controversial [9]. A key driver of resistance to anti-angiogenesis therapy has been identified as the increased expression of pro-angiogenic tumor-derived growth factors [10].

The chemokine (C-C motif) ligand 5 (CCL5; aka RANTES) is an 8kDa peptide that is up-regulated in breast tumors, and has been associated with metastatic spread [11-13]. CCL5 produced by mesenchymal stem cells (MSCs) acts to induce malignancy-associated changes in breast cancer cells to promote spread through binding with its cognate receptor, CCR5 [14]. In addition, recent work has also suggested a role for CCL5/CCR5 signaling in neovascularization, including as a key factor in EPC mediated angiogenesis [15]. For instance, wound healing has been shown to be CCR5 dependent [16, 17], while CCR5 antagonist administration results in reduced metastasis [18], and primary breast tumors that are less vascular (paler) than controls [19]. Although work from our laboratory and others has shown that CCR5 is expressed by EPCs [3] the mechanism of CCL5/CCR5 signaling in EPC mediated breast tumor neovascularization remains unclear.

Herein, using suppression of CCL5 in two immune competent murine breast cancer models, we demonstrate a paracrine role for cancer cell CCL5 in tumor neovascularization and growth. We further confirm expression of CCL5 receptors (CCR1 & CCR5) by EPCs, as well as significant tumor growth and angiogenesis defects in CCR5 null mice. However, ablation of CCR5 in the BM does not result in tumor vascular defects, while CCR5 null mice transplanted with wild type (WT) BM show tumor angiogenesis and growth defects, which phenocopy those observed in CCR5 null animals. This suggests that the defects observed in CCR5 null mice are not due to reduced numbers or functionality of EPCs. Instead, vascular defects in CCR5 null animals may be the result of defects in paracrine signaling between cancer cell CCL5 and vasculature resident in the tumor microenvironment. In support of this finding, we have identified a subpopulation of CCR5 expressing endothelial cells in the breast tumor microenvironment, and describe a clinical correlation between vascular expression of CCR5 and invasive tumor grade. We further demonstrate that suppression of endothelial CCL5/CCR5 signaling leads to defects in mTOR/AKT pathway activation [20], as well as vascular and tumor growth defects *in vitro* and *in vivo*.

## RESULTS

### Cancer cell produced CCL5 is required for tumor angiogenesis and growth

**Figure 1:**
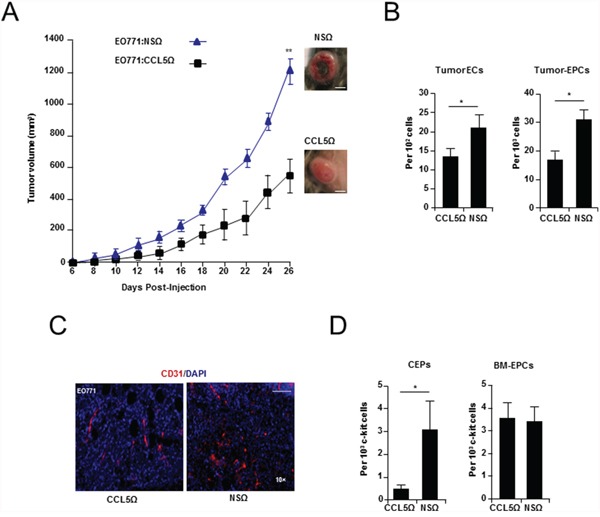
Cancer cell produced CCL5 is required for breast tumor growth and vascularization **A.** Growth curve of WT mice showing a significant reduction in the growth of EO771:EF_long_-eGFP-CCL5Ω (seed sequence 2), compared with EO771:EF_long_-eGFP-NSΩ tumors. Data was analyzed by MANOVA (α = 0.05, ‘^**^’*P* < 0.01, n = 10 per group). Tumor morphology as inset. Scale Bar, 10 mm. **B.** FACS analysis showing a significant reduction of tumor CD31^+^ CD11b^-^ endothelial cells (ECs) and tumor associated c-kit^+^ VEGFR2^+^ CD11b^-^ EPCs in EO771:CCL5Ω, compared with nonspecific control (EO771:NSΩ) tumors. Data is represented as mean number cells ± S.E.M. per 10^2^, or 10^3^ total cells (n = 5 per group). **C.** Immunostaining of vasculature (CD31^+^) from EO771:CCL5Ω and control tumors. Scale bar, 200 μm. **D.** FACS analysis showing a significant reduction in circulating endothelial progenitors (CEPs) (*Left*) and no significant difference in BM EPCs isolated from mice transplanted with EO771:CCL5Ω tumors (*Right*). Data is represented as either mean number per 1 × 10^3^ PB mononuclear cells (PBMNCs) or c-kit cells ± S.E.M. (n = 5 per group). For B & D, data was analyzed by Unpaired *t* test (‘^*^’*P* < 0.05, ‘^**^’*P* < 0.01; α = 0.05).

EO771 and 4T1 breast cancer cells were stably transduced with lentiviral vector (LVs) encoding CCL5 short hairpin RNA (generated with either seed sequence 2 or 3) and orthotopically implanted into mice (Supplementary Figure S1A-E and Supplementary Table S1A-S1G). Suppression of cancer cell CCL5 lead to significantly impaired tumor growth in both syngeneic mouse models (*P* < 0.001) (Figure 1A, Supplementary Figure S2A, S2B, and Supplementary Table S2A-S2C). When the tumors were examined, vascular defects were observed in size-matched tumors (Day 14). Such defects included a reduced number of endothelial cells (EO771: *P* = 0.0285 and 4T1: *P* < 0.0001), and a reduction in tumor localized EPCs (*P* = 0.0239) (Figure 1B, 1C, Supplementary Figure S2C and Supplementary Table S2D). Also evident, was a significant reduction in circulating EPCs (CEPs) (*P* = 0.033) (Figure 1D and Supplementary Table S2E), as well as a reduction in the number of BM resident EPCs (Figure 1D and Supplementary Table S2F). Thus, *in vivo* suppression of cancer cell CCL5 led to diminished tumor growth and vascular effects. However, as autocrine suppression of CCL5 did not result in significant proliferation defects *in vitro*, any effects on tumor growth and vasculature from suppression of cancer cell CCL5 are likely explained by paracrine signaling to the host microenvironment (Supplementary Figure S1F-S1G).

### Ablation of host CCR5 impairs tumor angiogenesis and growth

**Figure 2:**
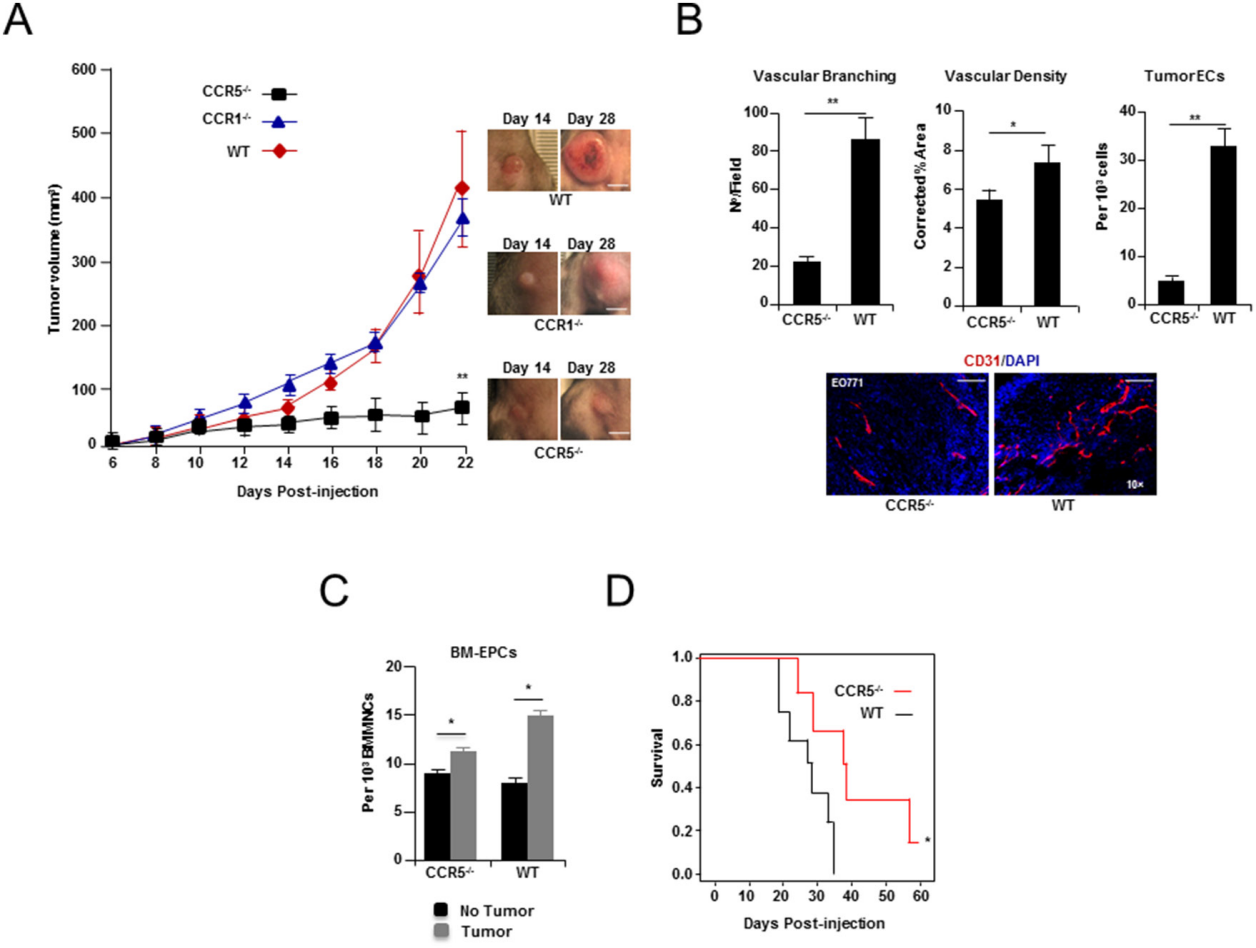
Breast tumor growth and angiogenesis in CCR5 null mice **A.** *Left*, growth curve of EO771 breast cancer cells showing a significant decrease in tumor growth when grown in CCR5^-/-^ null mice, compared with CCR1 null (CCR1^-/-^) or wild-type (WT) animals. Data is represented as mean volume ± S.E.M., and was analyzed by MANOVA (‘^**^’*P* < 0.01; α = 0.05, n = 10 per group). *Right*, Representative tumors (Day 14 & Day 28). Scale bar, 15 mm. **B.** *Upper Left*, shown, a significant decrease in tumor vascular branching in CCR5-/- null mice, compared with control (WT) mice. Data is represented as mean number of branch points/field ± S.E.M. (n = 20 per group). *Upper Middle*, shown, a significant decrease in tumor vascular density in CCR5-/- mice. Data is represented as mean CD31^+^ vasculature area/field, corrected for tumor area ± S.E.M. (n = 20 per group). *Upper Right*, FACS analysis of tumors showing a significant reduction in the number of CD31^+^ CD11b^-^endothelial cells (ECs) in CCR5^-/-^ mice, compared with WT mice. Data is represented as mean number of endothelial cells (ECs) per 10^3^ total cells ± S.E.M. *Lower*, CD31 immunostaining of representative tumors, showing significantly less vascular branching and density in CCR5^-/-^ mice compared with WT mice. Scale bar, 200μm. For B & C, data was analyzed by Unpaired *t* test (‘^*^’*P* < 0.05, ‘^**^’*P* < 0.01; α = 0.05). **C.** FACS analysis of bone marrow (BM) at day 14 from tumor challenged mice, showing expansion of VEGFR2^+^ c-kit^+^ CD11b^-^ EPCs in both WT (*P* = 0.0125), and CCR5-/- animals (*P* = 0.0478). Data is represented as mean number of cells per 10^3^ BM mononuclear cells (BMMNCs) ± S.E.M. (n = 5 per group). **D.** Survival data of CCR5^-/-^ and WT mice after tail vein injection of 1 × 10^5^ EO771 cells (n = 7) showing significantly increased survival in CCR5^-/-^mice. Data was analyzed by Kaplan-Meier estimator (‘^*^’*P* < 0.05, α = 0.05).

To assess the importance of paracrine signaling by cancer cell produced CCL5, EO771 cells were orthotopically injected into mice that were homozygous null for CCR1 (CCR1^-/-^), and/or CCR5 (CCR5^-/-^). Cancer cells grown orthotopically in the mammary gland of CCR5 null mice, but not CCR1 null mice, showed dramatically reduced tumor growth, compared to control wild-type (WT) animals (*P < 0.0001*) (Figure 2A and Supplementary Table S3A). Moreover, histological examination of size matched tumors that were harvested just prior to exponential growth (Day 14), revealed significant angiogenic defects in CCR5 null mice, including reduced vessel branching (*P* < 0.0001), decreased vascular density (*P* = 0.0273), and reduced tumor endothelial cell numbers (*P* = 0.0002) (Figure 2B and Supplementary Tables S3B-S3D). In agreement with previously published studies [2, 4], the number of EPCs in the BM of CCR5 null transgenic mice expanded in response to tumor challenge (*P* = 0.0478) (Figure 2C and Supplementary Table S3E).

Next, to determine whether ablation of CCR5 inhibits the propensity of breast cancer cells to establish lesions in the lungs, mCherry labelled [21] EO771 cells were injected into the tail-vein of CCR5^-/-^ mice. CCR5^-/-^ mice survived longer than control animals (*P* = 0.022) (Figure 2D, Supplementary Figure S3A, and Supplementary Table S4A), and metastases were less vascular, than those identified in control animals (*P* = 0.0037) (Supplementary Figure S3A-S3C and Supplementary Table S4B). Taken together the tumor growth and vascularization defects observed following ablation cancer cell of CCL5, and in CCR5 null mice, strongly support a role for tumor CCL5/host CCR5 paracrine signaling in tumor growth and neovascularization.

### CCR5 is not required for BM-mediated tumor growth and angiogenesis

**Figure 3:**
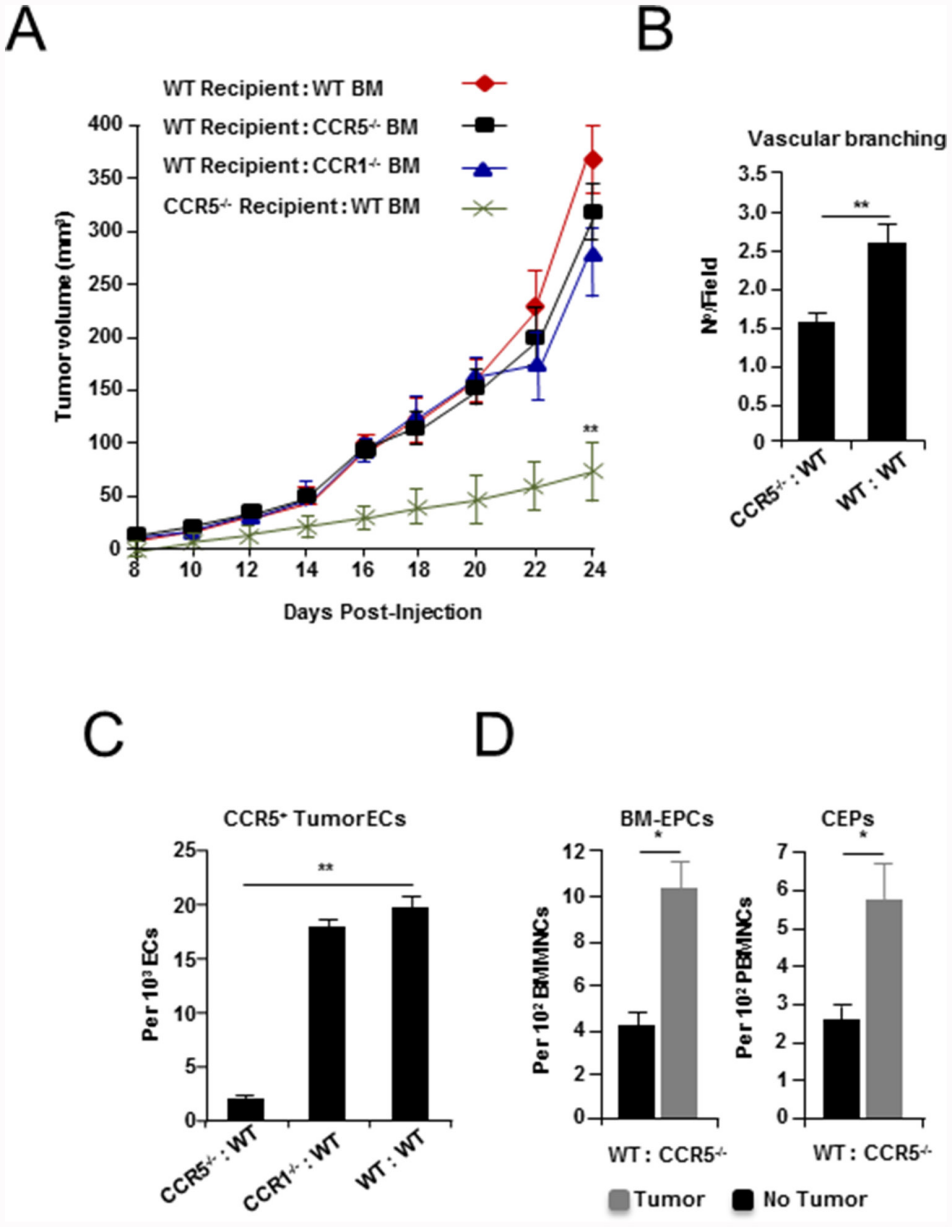
Tumor growth and angiogenesis in CCR5 null mice transplanted with wild-type (WT) bone marrow (BM) **A.** Growth curves of EO771 tumor cells grown in WT mice transplanted with WT, CCR1 null (CCR1^-/-^) and CCR5 null (CCR5^-/-^) BM (*recipient:donor*), as well as CCR5-/- mice transplanted with wild-type (WT) BM (CCR5^-/-^ :WT), showing a significant decrease in tumor growth in CCR5^-/-^:WT mice. Data is represented as mean tumor volume ± S.E.M. (n = 6), and analyzed by MANOVA (‘^**^’*P* < 0.01; α = 0.05). **B.** Shown a significant decrease in vascular branching in CCR5^-/-^:WT mice, compared with WT:WT animals. Data is represented as number of branch points/field ± S.E.M. (n = 20 per group). **C.** The number of CCR5^+^ CD31^+^ CD11b^-^ tumor endothelial cells (ECs) is significantly reduced in CCR5-/- transplanted with WT BM, compared with CCR1-/- transplanted with WT BM and control BMT mice. Data is represented as mean number of ECs per 10^3^ total CD31^+^ CD11b^-^ tumor ECs. **D.** FACS analysis of BM EPCs and peripheral blood (PB) CEPs from WT:CCR5^-/-^ animals, showing expansion of VEGFR2^+^ c-kit^+^ CD11b^-^ EPCs (*Left*), and CEPs (*Right*), after tumor challenge. Data is represented as mean number of EPCs (or CEPs) per 10^2^ BMMNCs/PBMNCs ± S.E.M. For B-D, data was analyzed by Unpaired *t* test (‘^*^’*P* < 0.05, ‘^**^’*P* < 0.01; α = 0.05).

Work by ourselves and others has shown that BM EPCs express both mRNA [3] and protein for CCR1 and CCR5 (Supplementary Figure S4A-S4D). Therefore, to determine the relative contribution of BM CCL5 receptor expression to tumor growth and neovascularization, BM from CCR1 or CCR5 null mice was transplanted into WT animals. For comparison, BM from WT mice was transplanted into CCR5 null animals (Supplementary Figure S5A). Following reconstitution, BM transplanted (BMT) animals were orthotopically implanted with EO771 breast cancer cells. Notably, tumor growth in WT mice transplanted with CCR1 null (WT:CCR1^-/-^), or CCR5 null BM (WT:CCR5^-/-^) did not significantly differ from tumors growing in WT mice reconstituted with WT BM (WT:WT). In contrast, EO771 tumors implanted in CCR5 null mice transplanted with WT BM (CCR5^-/-^:WT) showed significantly reduced growth (Figure 3A and Supplementary Table S5A), and a significant reduction in vessel branching (*P* < 0.0001), as well as a reduction (~50 %) in numbers of CCR5^+^ tumor-recruited endothelial cells (*P* < 0.0001) (Figure 3B, 3C, and Supplementary Table S5B, S5C).

The BM of BMT mice was then examined to determine whether defects in BM EPCs might explain the impaired growth and vascular defects observed. However, analysis of BM following reconstitution showed no significant difference in levels of EPCs, myeloid progenitors (MPs), or neutrophil progenitors (NPs) (Supplementary Figure S5B and Table S5D-S5G). Notably, no reduction in levels of CCR5^+^ BM EPCs was observed in CCR5^-/-^:WT BMT mice (Supplementary Figure S5C); while BM-EPCs and CEPs in WT:CCR5^-/-^ BMT animals responded normally to tumor challenge (Figure 3D). Furthermore, there was no observable reduction in the number of tumor recruited EPCs in animals with CCR5 null BM (Supplementary Figure S5D). Therefore, while absence of CCR5 in the non-BM compartment of the tumor-stroma lead to vascular defects, and a reduction in the number of tumor recruited CCR5+ endothelial cells, in the absence of BM CCR5 EPCs still proliferated and migrated to the blood and tumor-stroma. Taken together, these findings indicate that the vascular defects observed in CCR5 null mice are not the result of impaired EPCs biology, but instead, may be explained by defects in signaling to CCR5 expressing endothelial cells in the tumor microenvironment.

### Endothelial CCR5 expression is increased in response to tumor conditioned media and correlates with invasiveness in breast cancer

**Figure 4:**
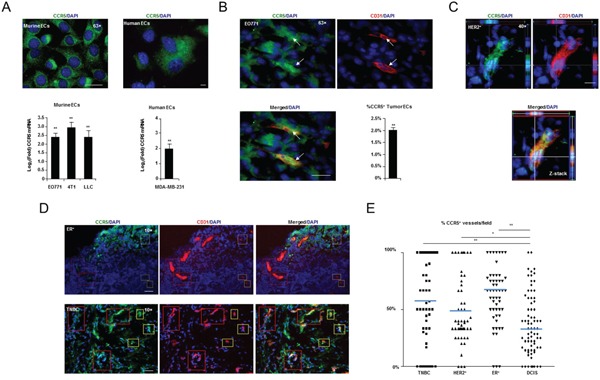
CCR5 expressing vasculature and pathology **A.** *Upper*, High resolution (63×) fluorescent microscopy showing CCR5 expression in murine (MHEVC) and human (HUVEC) endothelial cells (ECs) *in vitro*. Scale bar, 20 μm. *Lower*, Q-PCR showing significant induction of CCR5 mRNA in murine and human ECs, in response to murine (EO771, 4T1 & LLC) and human (MDA-MB-231) tumor conditioned medium, respectively. Data is represented as mean Log2(Fold) ± S.E.M. (n = 5 per group). **B.** High resolution (63×) fluorescent microscopy showing that CD31^+^ endothelial cells (ECs) express CCR5^+^ in EO771 tumors *in situ* (arrows). *Lower Right*, Results of FACS analysis showing the percentage of CD31^+^ CD11b^-^ ECs that are CCR5^+^ in EO771 tumors. Data is represented as a mean percentage of the total number of tumor ECs ± S.E.M. **C.** High resolution (40×) fluorescent microscopy Z-stack showing that CD31^+^ vasculature in human HER2^+^ breast cancers express CCR5. Scale bar, 10 μm. **D.** Fluorescent microscopy showing estrogen receptor (ER)^+^ (*Upper*) and triple negative (TNBC) (*Lower*) tumors, with CCR5 positive and negative vessels (red boxes) and ECs (yellow boxes). Scale bar, 50μm. **E.** Scoring analysis of CCR5 expression in CD31^+^ vasculature from TNBC, HER2^+^, ER^+^ and DCIS human breast cancers. Dot plot showing a significantly higher number of CCR5^+^ CD31^+^ tumor blood vessels in TNBC, HER2^+^ and ER^+^ tumors compared with DCIS. At least 10 fields were analyzed per section. For A, B & E, data was analyzed by Unpaired *t* test (‘^*^’*P* < 0.05, ‘^**^’*P* < 0.01; α = 0.05).

Mouse and human endothelial cells were treated with media pre-conditioned by exposure to mouse and human breast cancer cell lines. CCR5 mRNA was up-regulated, by at least two-fold (*P* < 0.05), in murine endothelial cells treated with pre-conditioned media from murine breast cancer cell lines (EO771, 4T1), and the highly angiogenic Lewis lung carcinoma line (LLCs). CCR5 mRNA was also up-regulated in human endothelial cells exposed to media conditioned by MDA-MB-231 human breast cancer cells (Figure 4A and Supplementary Table S6).

 When tumor vasculature was examined by immunofluorescence (IF) *in situ*, CCR5^+^ endothelial cells were identified in EO771, 4T1 and LLC tumors, with 2-20 % of tumor endothelial cells expressing CCR5 (Figure 4B and Supplementary Figure S6A, S6B). To account for vascular mimicry mCherry FACS exclusion was used (Supplementary Figure S7A, S7B) [22].

We next examined CCR5 vasculature in Triple Negative (TNBC), Human Epidermal Growth Factor Receptor 2 (HER2)^+^ and Estrogen Receptor (ER)^+^ breast cancers; as well as ductal carcinoma *in situ* (DCIS) [23]. In agreement with results of mouse studies, CCR5 was found to be expressed in a sub-population of human breast tumor vessels (Figure 4C, 4D). Interestingly, the number of CCR5^+^ endothelial cells, as a proportion of total vasculature, was higher in malignant tumors than in DCIS lesions: TNBC, *P* = 0.0017; HER2^+^, *P* = 0.0138; ER^+^, *P* < 0.0001 (Figure 4E and Supplementary Table S6B, S6C). Collectively, these findings indicate that vascular scoring with CCR5 in human tumors can be used as an indicator of malignancy, and that CCR5^+^ vasculature represents a clinically relevant sub-population in the tumor microenvironment.

### Suppression of CCL5/CCR5 leads to angiogenic defects *in vitro* and *in vivo*

**Figure 5:**
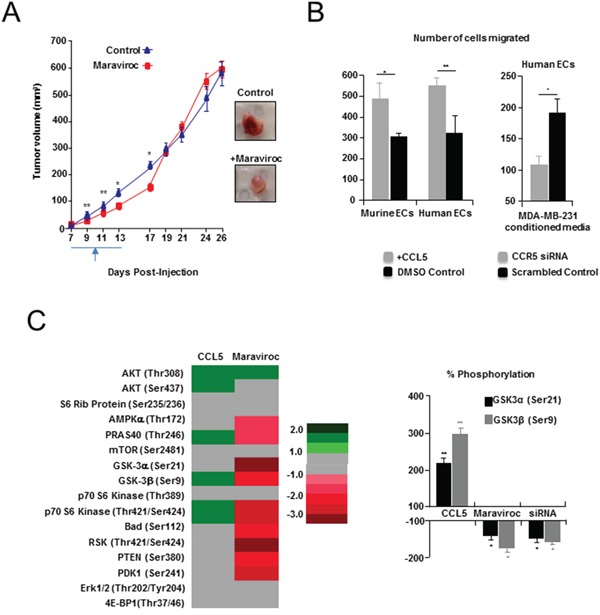
Pharmacologic inhibition of CCL5/CCR5 in a syngeneic model of breast cancer **A.** *Left*, delayed tumor growth, following maraviroc administration, compared with controls. Treatment commenced on day seven, and was followed by twice daily oral administration (gavage) of 10 mg/kg (3 % DMSO) maraviroc. Controls received vehicle only. *Right*, Tumors resected from maraviroc-treated animals (Day 13) were also paler. Scale bar, 10 mm. **B.** *Left*, Transwell assays showing significant number of mouse (MHEVS) and human (HUVEC) endothelial cells (ECs) migrated towards CCL5, compared to BSA control. Also shown (*Right*), a significant decrease in migration of human ECs in response to conditioned media from MDA-MB-231 breast cancer cells following siRNA suppression of CCR5, compared with scrambled siRNA control. Data is represented as mean number of migrated cells ± S.E.M. **C.** *Left*, Heat map of Pathscan AKT pathway analysis showing fold change in phosphorylation status of several members of the pathway in murine ECs, following treatment with CCL5 or suppression of CCR5 with maraviroc. Fold is determined by mean difference in average pixel density, between treated and control spots, following normalization. *Right*, Change in phosphorylation of GSK-3α (Ser21) and GSK-3β (Ser9) following treatment with CCL5, CCR5 siRNA or maraviroc, was confirmed by western blot analysis, and determined following normalisation against total GSK-3 protein. Tubulin was used as a loading reference. Data represented as mean % change in phosphorylation ± S.E.M. For B & C, data was analyzed by Unpaired *t* test (‘^*^’*P* < 0.05).

Next, murine and human endothelial cells treated with either siRNA inhibiting CCR5, or the CCR5 antagonist maraviroc, underwent endothelial tube formation assays *in vitro* (Supplementary Figure S8A-S8C and Supplementary Table S7A-S7C). Suppression of CCR5 led to significantly reduced tube length in endothelial cells (~10-30 % reduction). Maraviroc was then administered orally to mice implanted with mCherry labelled 4T1 tumor cells. Mice treated with maraviroc displayed delayed tumor growth for the period of treatment (Days 7-17; *P* < 0.05) (Figure 5A and Supplementary Table S8A). When maraviroc treatment ceased, tumor growth resumed and caught up with controls, although tumors were paler at harvest. Maraviroc-treated mice also showed no change in total weight or spleen weight compared with untreated animals (Supplementary Tables 8B,C). Histological examination conducted at end point (Day 28) revealed that treated tumors had a smaller growing margin (hematoxylin^+^) (Supplementary Figure S9A) and were less vascular (CD31^+^) than controls (Supplementary Figure S9B). In a parallel experiment, tumors were resected after treatment and mice allowed to develop metastases. In this case, tumor resected animals showed fewer numbers of circulating tumor cells (CTCs: *P* = 0.0461) [24] and fewer lung metastases (Supplementary Figure S9C, S9D and Supplementary Table S8D). This result suggested that angiogenesis is activated at least in part by signaling through CCR5.

To examine this pathway further we treated mouse and human endothelial cells, with CCL5, or tumor conditioned media, and demonstrated that migration is significantly impaired following siRNA suppression of CCR5 (Figure 5B and Supplementary Table S9A, S9B). We have also shown that while CCL5 treatment results in the phosphorylation/activation of downstream effectors of CCR5 activation; including members of the AKT/mTOR pathway (4EPBP1 & mTOR) [20]; suppression of CCR5 activation by maraviroc, or CCR5 siRNA treatment, lead to specific activation (phosphorylation) defects in members of this pathway (GSK-3α/GSK-3β) in murine endothelial cells (Figure 5C, Supplementary Figure S10A, S10B and Supplementary Table S10A, S10B).

Taken as a whole this work strongly suggests that CCL5 acts through CCR5 to promote angiogenesis, and that tumor neovascularization mediated by CCL5/CCR5 is not BM dependent. Furthermore, inhibition of mTOR/AKT signaling in endothelial cells may, at least in part, explain the anti-angiogenic effects observed following suppression of CCL5/CCR5 *in vitro* and *in vivo*.

## DISCUSSION

In previous work, CCL5/CCR5 antagonism has been shown to inhibit breast tumor metastasis [18], leading to tumors that are pale and necrotic [19]. This has implied that signaling through CCL5/CCR5 is needed for tumor vascularisation in breast cancer. However, previous work has been conducted in immune suppressed animals [18], making the contribution of cancer cell derived CCL5 to tumor angiogenesis in breast cancer difficult to assess. Moreover, Weinberg and others have proposed that CCL5 produced by mesenchymal stem cells (MSCs), directly acts on breast cancer cells to promote proliferation and spread [14]. Therefore, to determine the specific contribution of cancer cell derived CCL5 to angiogenesis we stably suppressed CCL5 in immune competent breast tumor models. In this work, we demonstrate that distinct tumor angiogenesis defects associated with suppression of cancer cell CCL5 are associated with a reduced number of tumor EPCs, and a reduction in the number of CEPs.

Next, to investigate the paracrine mechanism that cancer cell CCL5 operates to promote angiogenesis, we orthotopically implanted breast cancer cells into mice, which were homozygous null for either CCR1 or CCR5. At the start of the angiogenic switch (Day 14) tumors were significantly less vascular in CCR5 null animals than size matched tumors grown in CCR1 null or control animals. Further, the expected mobilization of EPCs into the peripheral blood in response to tumor challenge [2] was also impaired in CCR5 null animals. Delayed tumor growth and metastasis in CCR null mice has previously been reported by van Deventer and others [25, 26]. In our hands, tail vein injection of breast cancer cells also resulted in higher survival rates in CCR5 null animals. Notably, lung established in CCR5 null animals tail vein injected with breast cancer cells, were smaller and less vascular than those in WT animals.

As EPCs express both CCR1 and CCR5, it has been suggested that homing of EPCs to maturing vasculature is chemokine dependent [3, 15, 27]. Thus loss of CCR5 in EPCs may, at least in part, explain the tumor phenotype we observed in CCR5 null animals [17, 18]. Unexpectedly, we found that tumor growth and angiogenesis was not inhibited in wild-type mice transplanted with CCR5 null BM. Conversely, transplantation of wild-type BM into CCR5^-/-^ mice was not sufficient to rescue the reduced tumor growth and vascularization seen in CCR5 null mice, indicating that recruitment of CCR5^+^ BM-derived cell populations does not significantly contribute to the pro-angiogenic effects of CCL5/CCR5 signaling in breast tumors. This result is consistent with reports showing that transfer of CCR5^+^ BM cells in CCR5 null animals does not rescue tumor growth defects in melanoma [25, 26]. Additionally, as the number of BM EPCs was not significantly reduced in WT mice reconstituted with CCR5 null BM, the tumor growth and vascular phenotype associated with CCR5 ablation is likely due to a defect in the non-BM compartment of the tumor-microenvironment. Notably, while it has been shown that CCR5 plays a role in EPC tumor biology, this has only been demonstrated in the later stages of tumor development, and after rapid growth and angiogenesis associated with the angiogenic switch [15]. Furthermore, a proportion of tumor associated EPCs may not be of BM origin [2, 28]. For instance, two distinct adipose-derived EPC populations based on expression of c-c chemokine receptor-like 2 (CCRL2) have recently been identified [29]; although whether they play a role in the phenotype observed in CCR5 null animals remains unknown.

We have also shown that CCR5^+^ endothelial cells represent a distinct and clinically significant population in the breast tumor microenvironment, with a significant correlation between endothelial CCR5 expression and invasiveness. *In vitro*, endothelial cells treated with CCL5 also showed increased migration and activation of specific members of the AKT pathway, such as GSK3a/β [31]; whereas treatment with CCR5 antagonist or siRNA targeting CCR5 resulted in reduced migration and suppression of downstream effectors. Finally, we have shown that maraviroc treatment in an immune-competent mouse model of breast cancer, results in impaired metastasis and tumor growth. Taken as a whole, this work does not discount a role for other cell types in the indirect regulation of vascular biology. Instead findings support a mechanism by which cancer cell-derived CCL5 may directly recruit tumor vasculature from existing vasculature. In addition, while impaired vessel co-option [30] may offer an explanation for the defects observed following CCR5 deletion/suppression, in our hands inhibition of CCL5/CCR5 in endothelial cells resulted in specific mobilization defects *in vitro*. This finding supports supports the proposition that it is impaired recruitment of pre-existing ECs/vessels (angiogenesis), which best explains the vascular and tumor growth defects observed following CCR5 ablation or suppression *in vivo*.

In conclusion, specific therapies inhibiting CCL5/CCR5 may not only prevent malignant progression, but also significantly delay tumor growth by inhibiting the angiogenic switch in primary tumors and disseminated micrometastases. Given the unwanted side effects associated with generalised anti-angiogenesis therapies, directly inhibiting cancer cell CCL5 signaling to endothelial cells may constitute a novel strategy for blocking angiogenesis, tumor growth and spread in breast cancer.

## MATERIALS AND METHODS

### Cell culture and preparation of retroviruses

MDA-MB-231 cells were obtained from ATCC (Manassas, VA). E0771 and 4T1 cells were provided by Prof Robin Anderson (Olivia Newton John Cancer Research Institute, Australia). MDA-MB-231, 4T1 and EO771 cells were maintained in DMEM with 10% Fetal Calf Serum (FCS) (Invitrogen, Carlsbad, CA). EO771 and 4T1 cell lines expressing mCherry [21] were created through the stable transduction of a retroviral construct containing the mCherry gene [32]. EO771 and 4T1 cell lines with CCL5 KD were created through stable transduction of a retroviral construct containing shRNAs designed to inhibit CCL5 [4]. Four different seed sequences were created using RNAi Codex (codex.cshl.edu) [33]: (i) CCL5Ω(1): 5’-GAGAAGAAGTGGGTTCAAGAA-3’; (ii) CCL5Ω(2): 5’-CGACCAAGAAATCAGCATTTCATT-3’; (iii) CCL 5Ω(3): 5’-GGTTCAAGAATACATCAACTA-3’; and (iv) CCL5Ω(4): 5’-CGTGCCCACGTCAAGGAGTAT-3’. LLC/D122's were provided by L. Eisenbach (Wiesman Institute of Science, Rehovot, Israel), and maintained in RPMI with 10 % FCS. HUVECs were obtained from ATCC, grown on 0.1 % gelatin (Sigma-Aldrich), and maintained in EGM-2MV BulletKit™ media (Lonza, Valais, Switzerland). MHEVCs were provided by J. Cook-Mills (University of Cincinnati, Cincinnati, OH) [34], and maintained in RPMI with 10 % FCS. Cell authentication was conducted by short tandem repeat profiling, cell morphology monitoring, karyotyping, and the ATCC cytochrome c oxidase.

Lentivirus (LV) particles pseudotyped with the vesicular stomatitis G protein (VSVG), were generated by calcium phosphate transfection of three packaging constructs, pSPAX (REV/RRE) and pVSVG into human embryonic kidney (HEK) 293T cells [32]. Viral titer was determined by FACS analysis of LV-infected 293T cells. LV transductions of cell lines were performed in the presence of Polybrene^®^ (Sigma-Aldrich). Cells transduced with PGK-mCherry were assessed for their similarity in growth and pathology to the parental line. 293T cells were obtained from ATCC and grown in DMEM, with 10 % FCS, and Sodium Pyruvate (1 mM).

### Tumor growth and bone marrow transplantation (BMT) studies

Female C57BL/6 mice and BALB/c mice and were obtained from the Animal Resources Centre (Canning Vale, Western Australia). All procedures involving mice were conducted in accordance with protocols reviewed and approved by institutional animal care and ethics committees. Mice homozygous null for CCR1 (B6.129S4-*Ccr1^tm1Gao^*) and CCR5 (B6.129P2-*Ccr5^tm1Kuz^*/J) were sourced from The Jackson Laboratory, Bar Harbor, MN) [35, 36]. BMT was conducted according to previously published protocols [4]. In this study, 1 × 10^7^ total Wild-type (WT), CCR1^-/-^, or CCR5^-/-^ total BM cells were injected into the tail veins of lethally irradiated (1100 rads) C57BL/6 mice, CCR1 null (^-/-^), and or CCR5 null (^-/-^) recipients. Mice were used in tumor growth studies following reconstitution (8 weeks). Unless otherwise stated, mice were injected with 5 × 10^4^ breast cancer cells, orthotopically in the mammary fat pad of either C57BL/6 (EO771) or BALB/c (4T1) mice. Tumor size was measured using standard methods, and volume calculated using the following equation: (short axis)^2^ × (long axis) × 0.5236 [2, 4].

### Immunohistochemistry

Mouse tissues were fixed (4% PFA), and cryopreserved in optimal cutting temperature (O.C.T.) medium (Tissue-Tek, Elkhart, IN), and prepared as 10-30 μm thick sections. Mononuclear cells from the blood and BM were isolated by gradient centrifugation using Histopaque-1077 (Sigma-Aldrich, Milwaukee, WI) and centrifuged onto Superfrost Plus Slides (Menzel-Glaser, Braunschweig, Germany). Unless otherwise stated, all tissues were stained with Alexa Fluor^®^ (Invitrogen), or Phycoerythrin (PE) conjugated primary antibodies, as well as with DAPI. Rat or hamster anti-mouse primary antibodies: CD31/PECAM-1 (clone MEC13.3), VE-Cadherin/CD144 (clone 11D4.1), CD11b (clone M1/70), VEGFR2/Flk1 (clone avas12α1), CCR5/CD195 (clone C34-3448), c-kit/CD117 (clone 2B8), Ly-6G and Ly-6C/Gr-1 (clone RB6-8C5), CD4 (clone RM4-5) and CD61/Integrinβ3 (clone 2C9.G2) were obtained from BD Biosciences. We also used CCR1/CD191 (clone C-20), reactive against mouse and human, were Santa Cruz Biotechnology (Santa Cruz, CA). Rat Anti-Mouse Ki-67 eFluor^®^ 570 (SolA15) was purchased from eBioscience [4].

Formalin-fixed human breast cancer biopsies cryopreserved in O.C.T were provided by the Breast Cancer Tissue Bank (www.abctb.org.au). Biopsies were collected by Westmead Hospital, NSW, Australia under protocols reviewed and approved by institutional human ethics committees. In this study, biopsies were prepared as 10 μm thick transverse sections, prior to immunofluorescence analysis. For staining of human tissues we used mouse monoclonal anti-CCR5 (D-6) from Santa Cruz Biotechnology, and mouse anti-human CD31 (clone WM59) fromBD Biosciences. Images were obtained using the Zeiss M2 fluorescent microscope (Software Axiovision Version 4.8, Carl Zeiss, Aalen, Germany), as described [2-4].

### Flow cytometry

For FACS, cell suspensions were filtered (70 μm), pre-blocked with F_c_ block CD16/CD32 (BD Biosciences), and incubated with Alexa-dye, phycoerythrin (PE), or Allophycocyanin (APC) conjugated primary monoclonal antibodies. A rat monoclonal anti-mouse TER-119 (clone TER-119) antibody (BD Biosciences) was used as an erythroid marker [2-4]. Isotype, fluorescent minus one (FMO), and unstained controls were all used for determining appropriate gates, voltages, and compensation [37], using the BD Fortessa LSRII flow cytometer with FACS Diva software (BD Biosciences).

For FACS analysis we have defined EPCs as CD11b^-^ VEGFR2^+^ VE-cadherin^+^ c-kit^+^ and Ter119^-^, myeloid progenitors as CD11b^+^ c-kit^+^ Ter-119^-^, neutrophil progenitors as GR1^+^ CD11b^+^ c-kit^+^, and endothelial cells as CD11b^-^ CD31^+^. Because of relative marker levels in different tissues, tumor EPCs are specifically isolated as VE-cadherin^+^ CD11b^-^ c-kit^+^ Ter-119^-^ cells, while in the blood and BM VEGFR2^+^ c-kit^+^ CD11b^-^ Ter-119^-^ cells [2, 4, 27] (Supplementary Figure S11). Analysis of tumor, BM and blood EPC populations was conducted prior to the angiogenic switch (~Day 14 for EO771s) from mice sacrificed at this time.

### Tubule formation, wound healing and migration assays

For tube formation endothelial cells were grown on Matrigel™ (BD Biosciences, following standard methods [27]. Tube number and length were analyzed from randomly selected fields. For wound healing a scratch (wound) was made across a cell monolayer using a sterile tip. Image analysis was conducted using ImageJ software. Relative clearance rate was determined using the equation: (distance_=0h_ – distance_=24h_)/ distance_=0h_ × 100 % [27]. For migration assays, we used a transwell 8 μm polycarbonate membrane assay (Corning, Tewksbury, MA), with either conditioned media, or recombinant purified CCL5/RANTES (10 nM) (PreproTech, Rocky Hill, NJ) as chemoattractant. After four hours membranes were imaged using the IX71 fluorescent microscope and counted using CellSens Dimensions software (Olympus, Tokyo Japan).

Endothelial cells were transfected with siRNAs (0.04 pmol/μl) using siPORT NeoFX Transfection Agent (Ambion, Carlsbad, CA) [27], and/or treated with the small molecular inhibitor of CCR5, maraviroc (100 nM). Pooled siRNAs specifically designed to inhibit mouse or human CCR5 (siGENOME SMARTpool) and Cyanine 3 (Cy3) labeled siGLO RISC-Free Control siRNA were obtained from Thermo Scientific (Waltham, MA).

### Quantitative protein analysis

A CCL5 enzyme linked immunosorbent assay (ELISA) was used to quantitatively assess protein levels following CCL5 shRNAi transduction. ELISA followed the mouse CCL5/RANTES DuoSet protocol (R&D Systems, Minneapolis, MN). Microtitre plates were coated with CCL5-specific capture antibody overnight, washed three times and blocked for 1 hour with 1 % bovine serum albumin (BSA) in phosphate buffered saline (PBS). Detection antibody was goat anti-mouse CCL5 (R&D Systems). A standard curve was prepared using CCL5 protein by serial dilution with the colorimetric density of each well measured (450 nm). Duplicate assays were performed and results presented as the mean protein concentration (pg/ml) either secreted, or cell bound.

The PathScan® Akt Signaling Antibody Array Kit was conducted with fresh lysate (Cell Signaling Technology, Danvers, MA). After treatment with LumiGLO^®^, the chemiluminescence readout was visualised using the ChemiDoc XRS^+^ Imaging System and quantified using the Image Lab Software, Version 2.0.1 (Bio-Rad). Western blots were performed on maraviroc and CCL5 treated (10 nM) endothelial cell lysate using GSK-3 Antibody Sampler kit (1:1000, No: 9369, Cell Signalling Technology, Danvers, MA) and mTOR substrates Antibody Sampler kit (1:1000, No:9862, Cell Signalling Technology, Danvers, MA). Anti-CCR5 antibody (1:1000 dilution; M-20, Santa Cruz) and the house keeping gene α/β-Tubulin (1:1000, No:2148, Cell signaling), was also used. Primary antibody was detected with horseradish peroxidase (HRP)–anti-mouse IgG at 1:2000 (No:7074, Cell Signalling). Blots were developed with WestPico Supersignal (Pierce Biosciences, Rockford, IL) and chemiluminescence recorded using either the ChemiDoc XRS system (Bio-Rad Hercules, CA), or Fujifilm LAS 4000 image analyser.

### mRNA expression analysis

Q-PCR analysis using SYBR Green I was performed on the Rotor-Gene Q 2plex (Qiagen, Germantown, MD). Primers used for murine CCL5: *forward*: 5’-TACCATGAAGATCTCTGCAGCT and *reverse:* 5’-CTGCTGGTGTAGAAATACTCCT-3’. Levels of mRNA were normalized to 18S RNA using primers: *forward*: 5’-CTTAGAGGGACAAGTGGCG-3’ & *reverse*: 5’-ACGCTGAGCCAGTCAGTGTA-3’. Primers used for murine CCR5 (*forward*: 5’-CTGGACTCCCTACAACA TTG -3’ and *reverse*: 5’- ACACTGAGAGATAACTCCG G-3’); and human CCR5 (*forward*: 5’-CTGGGCTCCCTA CAACATTG-3’ and *reverse*: 5’-TGCAGGTGACAGAG ACTCTTG-3’).

### Statistical & data analyses

Statistical analyses were performed using Prism™ version 3.0 (GraphPad, La Jolla, CA). One-way MANOVA (α = 0.05) was used to compare differences in tumor growth. For comparison of the different cell populations following FACS Unpaired *t* test analysis was used (α = 0.05). Quantitative differences are represented as Log_2_(Fold) values (or ΔΔCT), and significance was determined through comparison of the difference in the two of ΔCT values, using Unpaired *t* test (α = 0.05) [38]. For vascular scoring (vascular branch point & vascular density analysis) images were imported into ImageJ software (v1.44, National Institutes of Health, Bethesda, MD). The total vascular density was calculated as a percentage of total tumor area, and branch points were counted by tallying each bifurcation point. Unless otherwise stated Unpaired *t* test analysis was applied to vascular scoring data, as well as data collected following analysis of tube formation and wound healing (α = 0.05).

## SUPPLEMENTARY MATERIALS FIGURES AND TABLES










