# Supplementary material for: Cancer cell CCL5 mediates bone marrow independent angiogenesis in breast cancer
Source: Oncotarget. 2016 Nov 16;7(51):85437–49. doi: 10.18632/oncotarget.13387 (PMC5356747; doi:10.18632/oncotarget.13387)
Supplement: Supplementary file 1 [file oncotarget-07-85437-s001.pdf]

Cancer cell CCL5 mediates bone marrow independent angiogenesis in breast cancer

SUPPLEMENTARY FIGURES AND TABLES

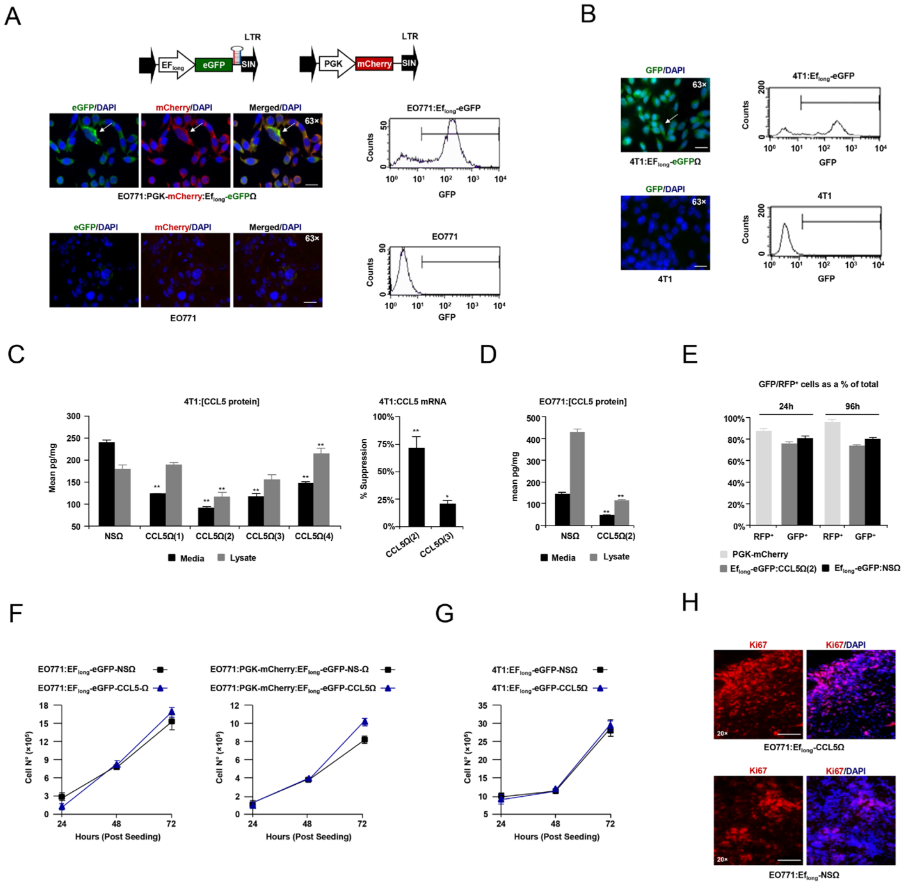

Supplementary Figure S1: Screening of CCL5 shRNAi. A. *Upper Left*, Schematic of LV construct containing short hairpin (Ω) RNA, targeting murine CCL5, or nonspecific (NS) seed sequence in a miR-30 based context. Also shown, Self Inactivating (SIN) LTRs, as well as eGFP and shRNAi driven by the elongation factor (EF) 1α1 promoter long (LV-EFlong-eGFP-Ω). *Upper Right*, Schematic of LV construct used to generate cells stably expressing mCherry driven by the constitutive PGK promoter (LV-PGK-mCherry), showing SIN LTRs. *Bottom Left*, Fluorescent microscopy *in vitro*, showing co-expression of GFP and mCherry in EO771 cells transduced with LV:eGFP-Ω, and LV:PGK-mCherry (white arrow), with no signal in non-transduced cells. Scale Bar, ~20 μm. *Bottom Right*, FACS showing expression of GFP in EO771:eGFP-Ω transduced cells, with no signal in non-transduced cells. **B.** *Left*, Fluorescent microscopy *in vitro*, showing expression of GFP in 4T1 cells transduced with LV:eGFP-Ω (white arrow), with no signal in non-transduced cells. Scale Bar, 20 μm. *Right*, FACS analysis showing expression of GFP in 4T1 cells transfected with LV:eGFP-Ω, with no signal in non-transduced cells. **C.** *Left*, ELISA showing significant reduction in level of media and lysate CCL5 protein in 4T1 cells stably transduced with LV:eGFP-CCR5Ω containing four different seed sequences (1-4), compared with LV:eGFP-NSΩ control, with the most reduction observed with seed sequence 2 [4T1:eGFP-CCL5Ω(2)]. Data is represented as mean p/μg ± S.E.M. (n = 3). *Right*, Q-PCR analysis showing a ~73 % reduction of CCL5 mRNA levels in 4T1:eGFP-CCR5Ω(2), and ~23 % reduction in 4T1:eGFP-CCL5Ω(3) compared with 4T1:eGFP-NSΩ control. Data is represented as the mean percentage reduction in mRNA ± S.E.M. **D.** ELISA showing a significant reduction in media and lysate CCL5 protein levels from EO771:eGFP-CCR5Ω (seed sequence 2), compared with EO771:eGFP-NSΩ control. Data is represented as mean pg/μg ± SEM (n = 3). **E.** Transduced constructs were stable with no difference in GFP^{+}/$mCherry^{+}$ signal in EO771:mCherry, EO771:eGFP-CCL5Ω(2) or EO771:eGFP-NSΩ after 24 and 96 hours. Data is represented as mean number of GFP/RFP^{+} cells as a percentage of the total number ± SEM. For C-E, data was analyzed by Unpaired *t* test ('*'$P < 0.05$, '**'$P < 0.01$; α = 0.05). Also shown, LV-CCL5Ω EO771 **F.** and 4T1 **G.** cells *in vitro* show no significant difference in proliferation, compared to NSΩ controls. No difference in Ki-67 staining **H**. For F & G, data was analyzed by MANOVA ('*'$P < 0.05$, '**'$P < 0.01$; α = 0.05).

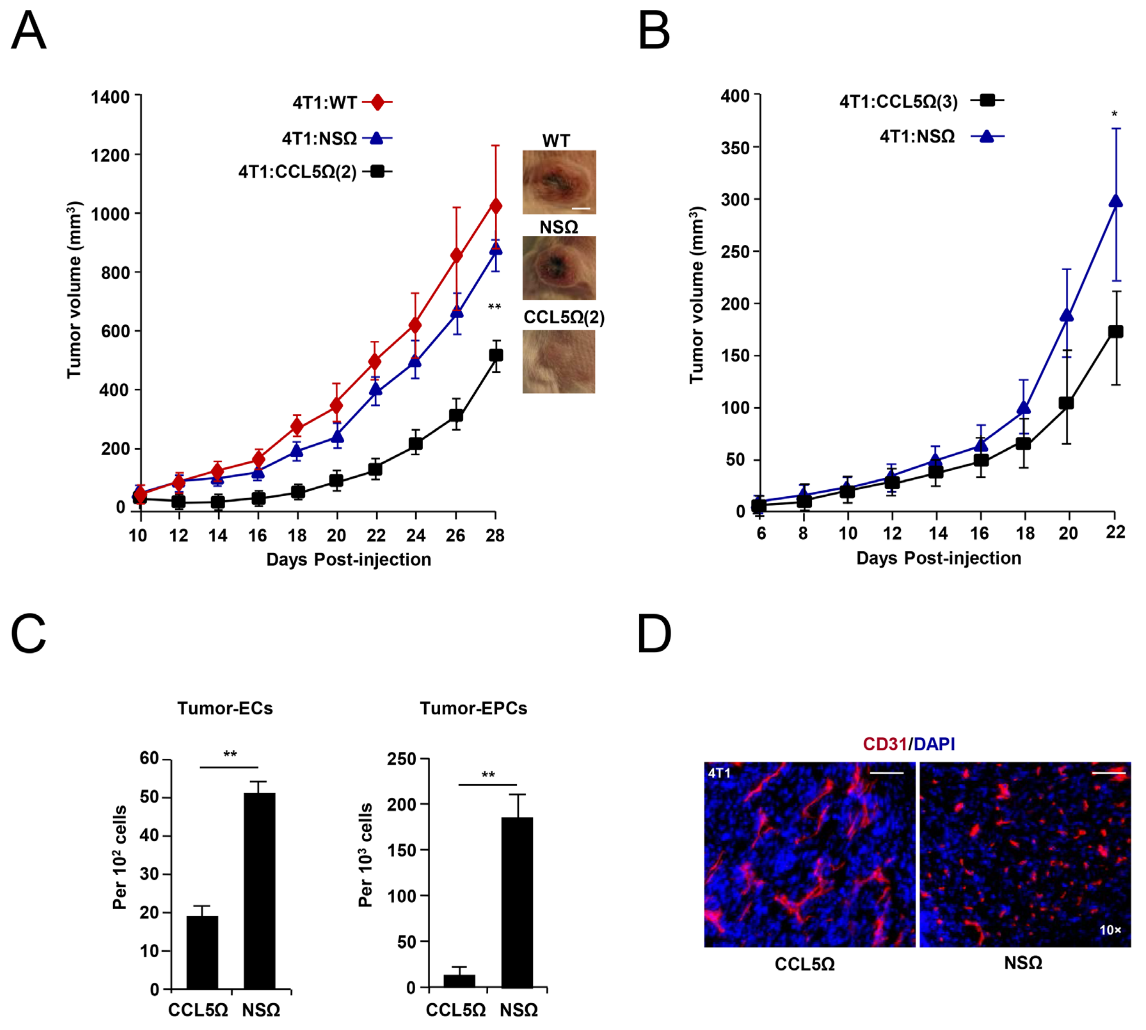

Supplementary Figure S2: CCL5 shRNAi in 4T1 breast cancer cells. A. Shown, a significant reduction in the growth of 4T1:EFlong-eGFP-CCL5Ω (seed sequence 2) compared with 4T1:EFlong-eGFP-NSΩ and 4T1 control tumors. Morphology shown as inset. Scale bar, 10 mm. **B.** Shown, a significant reduction in the growth of 4T1:eGFP-CCL5Ω (seed sequence 3) compared with control 4T1:eGFP-NSΩ tumors. *For A & B*, data is represented as mean volume ± S.E.M. (n=15), and analyzed by MANOVA (‘*’$P < 0.05$; $\alpha = 0.05$). **C.** *Left*, There is a significant reduction in number of ($CD31^+$ $CD11b^-$) tumor endothelial cells and ($CD31^{low}$, c-kit^+ $VEGFR2^+$ $CD11b^-$) tumor EPCs in 4T1:eGFP-CCL5Ω tumors, compared with 4T1:eGFP-NSΩ control tumors (‘**’$P = 0.0285$ & $P = 0.0239$, respectively; $\alpha = 0.05$). Data is represented as mean number of cells per total number of tumor cells ± S.E.M., and was analyzed by Unpaired t test (‘**’$P < 0.01$; $\alpha = 0.05$). **D.** $CD31^+$ immunostaining of 4T1:eGFP- CCL5Ω tumors, compared with 4T1:eGFP-NSΩ tumors. Scale bar, 200 μm.

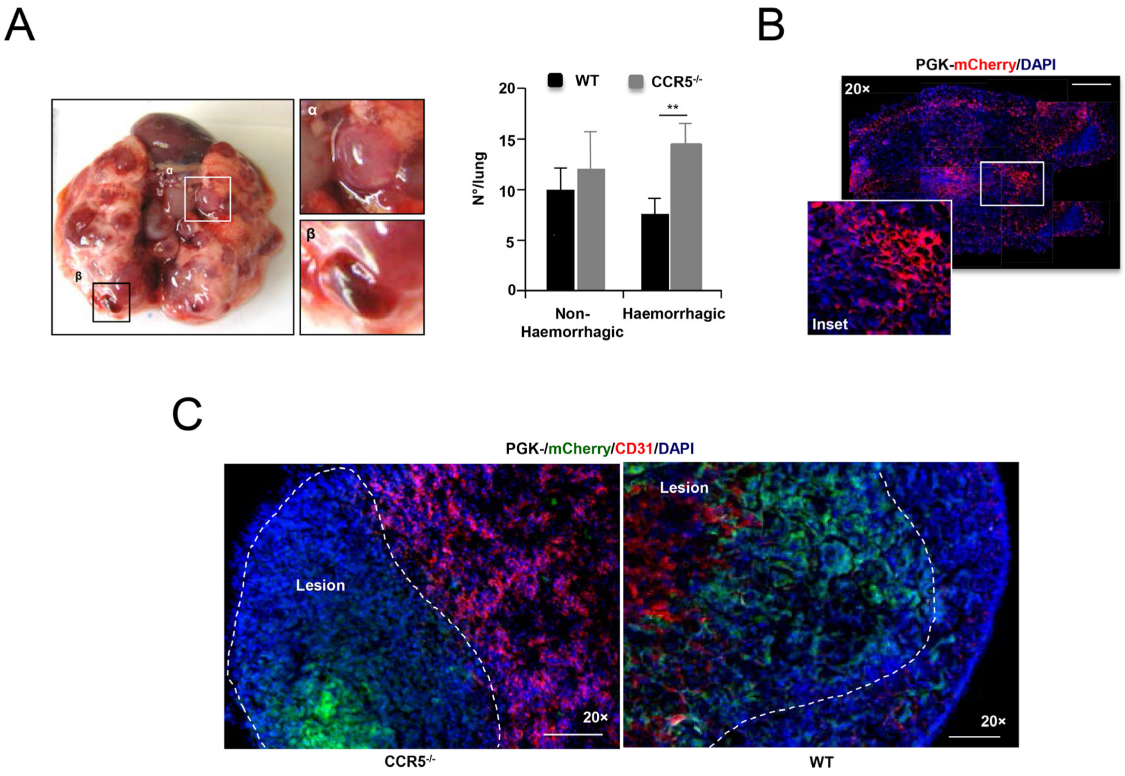

Supplementary Figure S3: Mouse model of lung metastasis. A. Representative image of metastases-bearing lungs after tail vein injection of EO771 breast cancer cells, showing non-haemorrhagic (α) and (β) haemorrhagic lesions. *Right*, Significant increase in haemorrhagic lesions by gross morphology in CCR5 null ($CCR5^{-/-}$) animals. Data is represented as number of lesions/lung and was analyzed by Unpaired *t* test (‘*’$P < 0.05$; $\alpha = 0.05$, n = 15). **B.** Representative image (combined tiles) showing $mCherry^{+}$ lesions in lungs from wild-type (WT) mice injected with PGK-$Cherry^{+}$ EO771 cells, via the tail vein. Scale bar, 500 µm. **C.** Fluorescent microscopy of metastasis bearing lungs from mice tail vein injected with PGK-$Cherry^{+}$ EO771 cells showing significantly less $CD31^{+}$ vasculature and invasive lesions in $CCR5^{-/-}$ mice (*Left*) compared with wild-type animals (*Right*). Scale Bar, 200 µm.

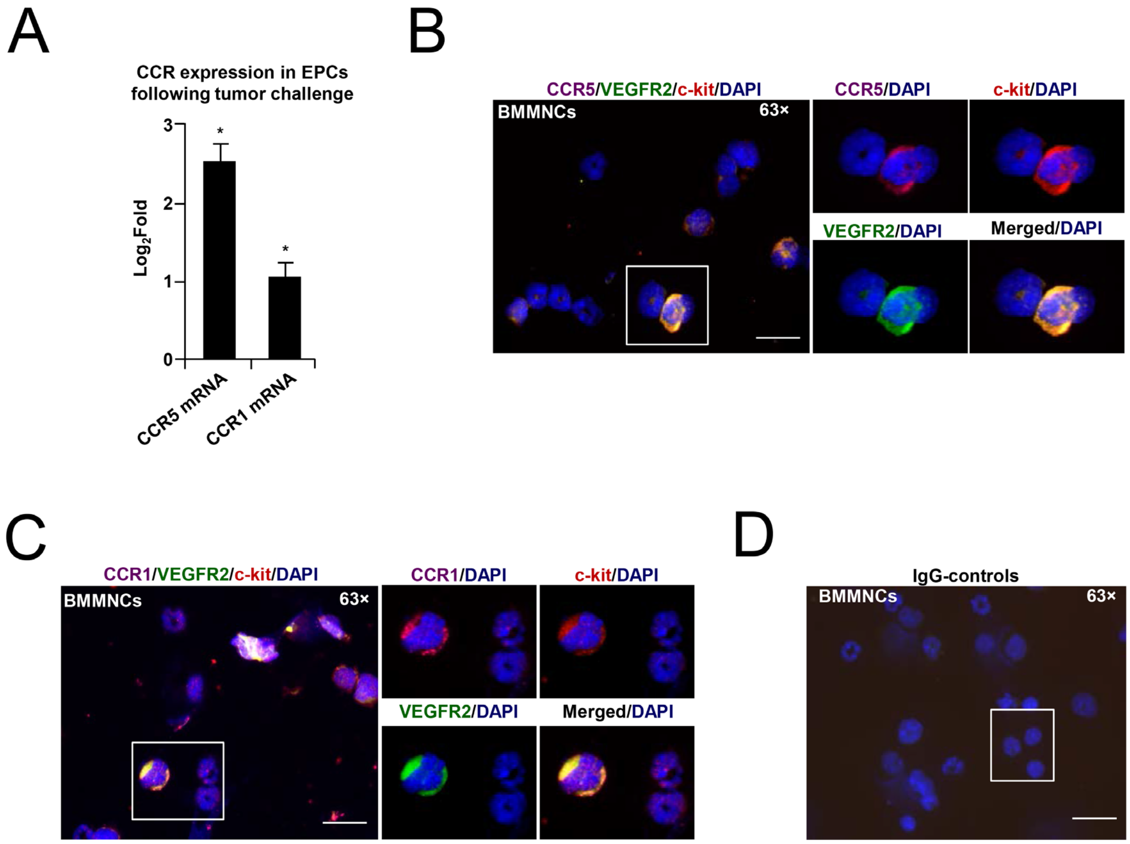

Supplementary Figure S4: EPCs express CCR1 and CCR5. A. Q-PCR analysis showing increased expression of CCR1 and CCR5 mRNA in tumor-EPCs, compared with bone marrow (BM)-EPCs. Data is represented as mean Log_2(Fold) ± S.E.M. Data was analyzed by Unpaired *t* test ('*' $P < 0.05$; $\alpha = 0.05$). **B.** High Resolution (63×) fluorescence microscopy of cytospun BM mononuclear cells (BMMNCs), showing expression of CCR5 in VEGFR2$^+$ c-kit$^+$ EPCs, with CCR5$^+$ EPC as inset. **C.** High Resolution (63×) microscopy of cytospun BMMNCs, showing expression of CCR1 in VEGFR2$^+$ c-kit$^+$ EPCs, with CCR1$^+$ EPC as inset. **D.** High Resolution (63×) microscopy of the negative (IgG) control showing no signal. For B-D, Scale Bar, 50 µm.

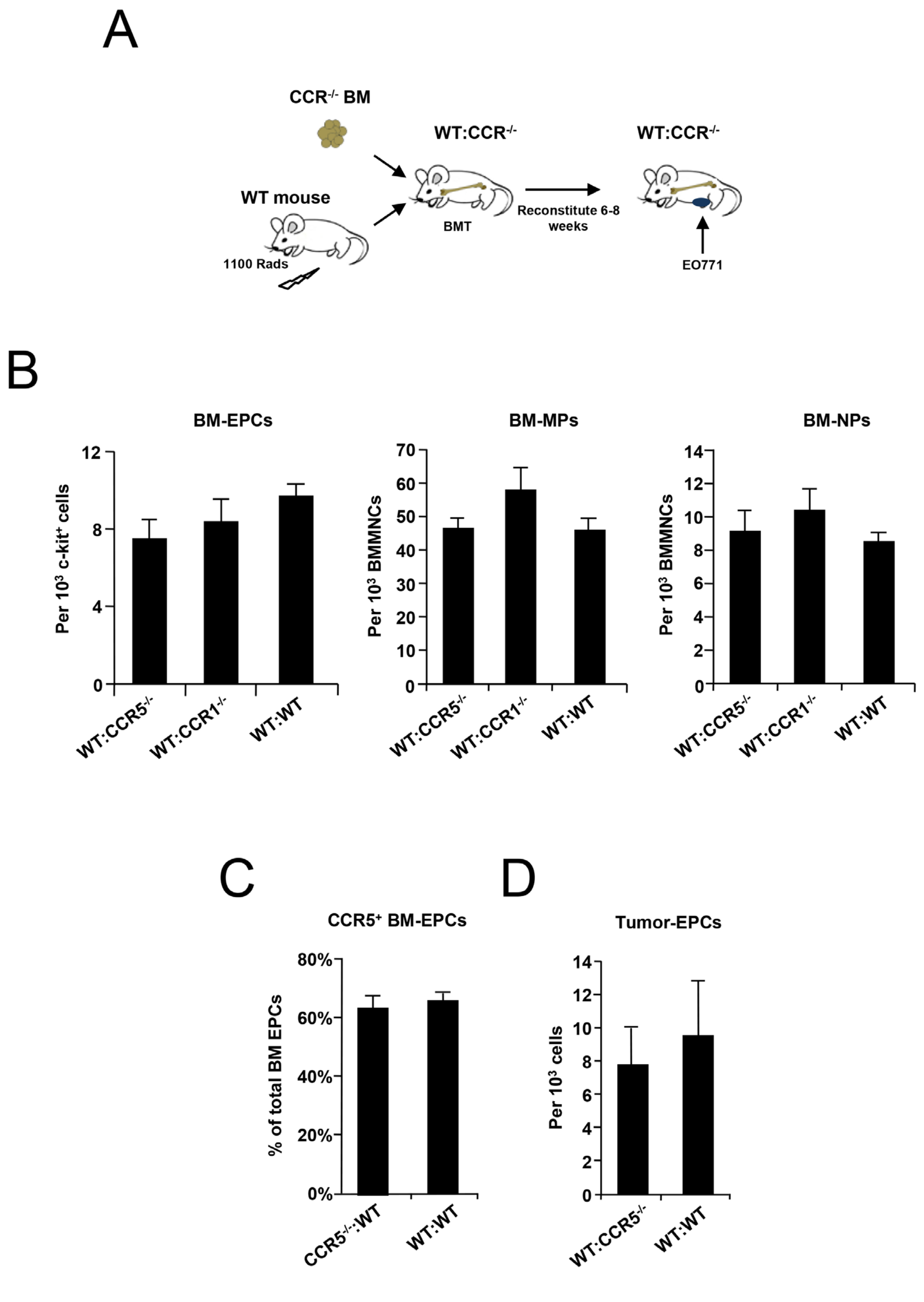

Supplementary Figure S5: Results of bone marrow (BM) transplantation. **A.** Schematic of the generation of CCR null (CCR$^{-/-}$) wild-type (WT) and WT:CCR$^{-/-}$ bone marrow (BM) transplanted (BMT) mice. C57BL/6 WT mice were irradiated (1100 rads) and transplanted with either total WT, CCR1$^{-/-}$ or CCR5$^{-/-}$ BM. **B.** Results of FACS analysis of BM from WT:CCR5$^{-/-}$, WT:CCR1$^{-/-}$ and WT:WT BMT animals, showing no significant difference in levels of VEGFR2^{+}, CD11b^{-}, c-kit^{+} EPCs, CD11b^{+} c-kit^{+} myeloid progenitors (MPs), or GR1^{+} c-kit^{+} neutrophil progenitors (NPs) after reconstitution. Data is represented as mean number of cells per 10^3 bone marrow mononuclear cells (BMMNCs), or c-kit^{+} BMMNCs ± S.E.M. **C.** Results of FACS analysis of BM from mice with EO771 tumors showing no difference in levels of CCR5^{+} VEGFR2^{+} CD11b^{-} EPCs isolated from CCR5$^{-/-}$:WT animals, compared with WT:WT controls, as a percentage of total VEGFR2^{+} CD11b^{-} EPCs. **D.** Shown, no significant difference in levels of tumor recruited VE-Cadherin^{+} CD11b^{-} EPCs in WT:CCR5$^{-/-}$ animals compared with control. Data is represented as mean number of EPCs per 10^3 cells ± S.E.M. Data is represented as a percentage of total BM resident EPCs. For B-D, data was analyzed by Unpaired *t* test (‘*’$P < 0.05$, ‘**’$P < 0.01$; $\alpha = 0.05$).

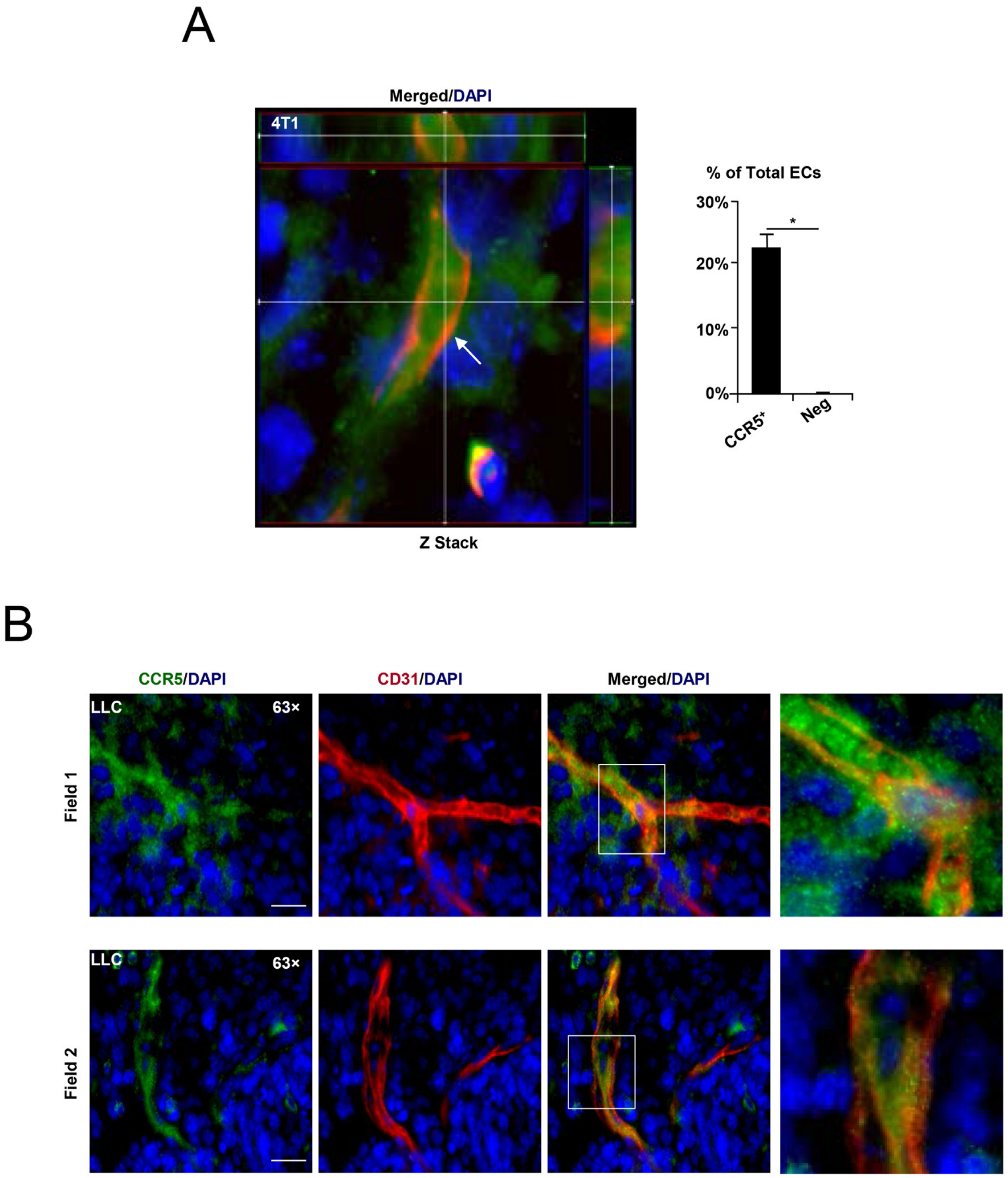

Supplementary Figure S6: Expression of CCR5 in tumor vasculature. A. *Left*, High resolution (63×) transverse immunofluorescence (IF) Z-Stack of 4T1 tumor $CD31^+$ vasculature showing CCR5 expression to be cytoplasmic. *Right*, FACS analysis showing the percentage of total 4T1 endothelial cells ($CD31^+$ $CD11b^-$) that are $CCR5^+$. Data represented as mean percentage of total tumor endothelial cells ± S.E.M., analyzed by Unpaired *t* test (‘*’$P < 0.05$, ‘**’$P < 0.01$; $\alpha = 0.05$). **B.** High resolution (63×) IF microscopy of LLC tumor (2 fields) showing CCR5 expression in tumor ($CD31^+$) vasculature. Scale bar, 20 µm.

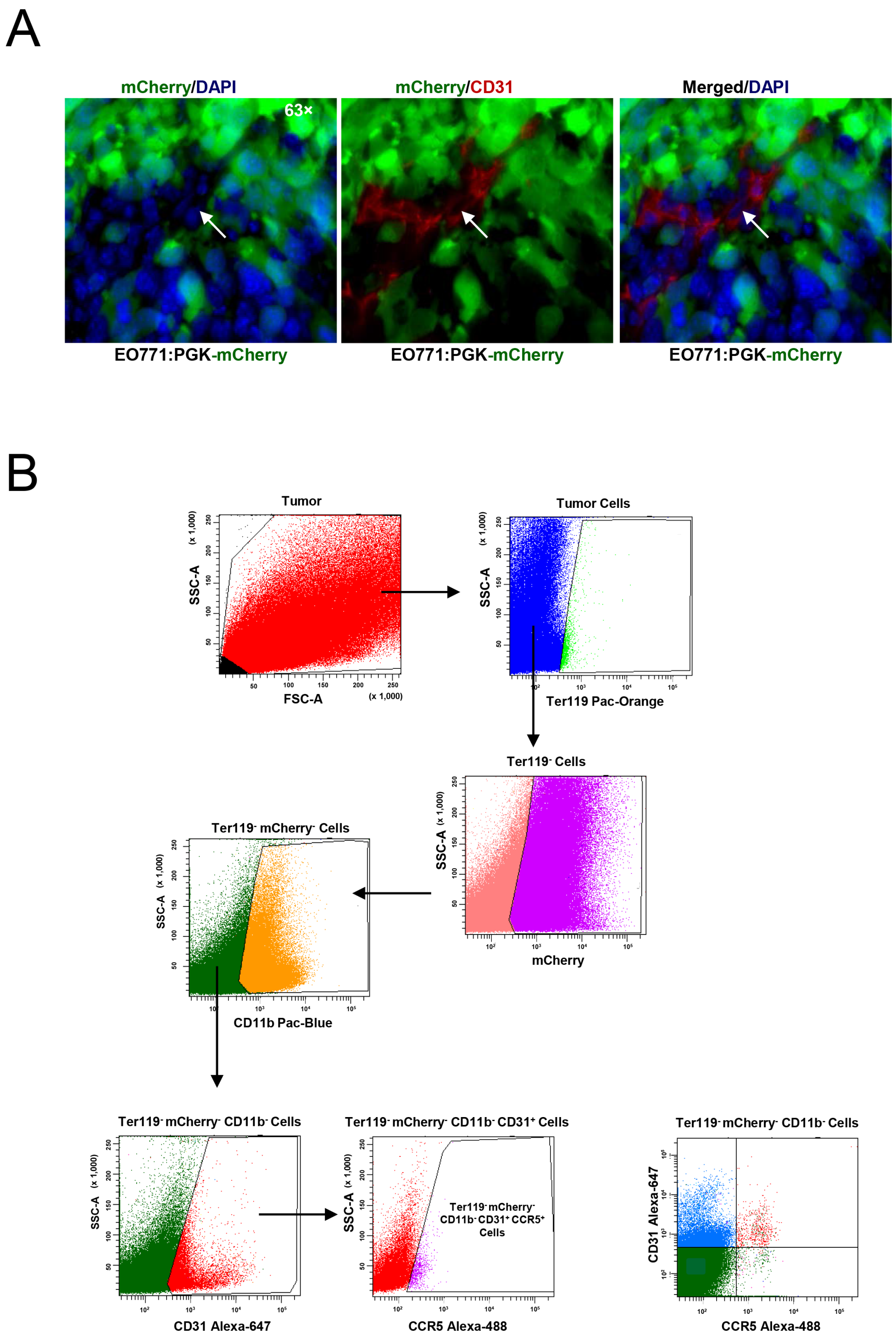

Supplementary Figure S7: Isolation of CCR5^{+} endothelial cells from the tumor microenvironment. A. Fluorescence microscopy of EO771:PGK-mCherry-labeled tumors showing separation of CD31^{+} mCherry^{-} vasculature from mCherry^{+} tumor cells, with inset of regions of interest. Scale Bar, 100 µm. **B.** Representative scatter plot showing FACS analysis of CCR5^{+} CD31^{+} EO771 endothelial cells following exclusion of TER-119, mCherry and CD11b.

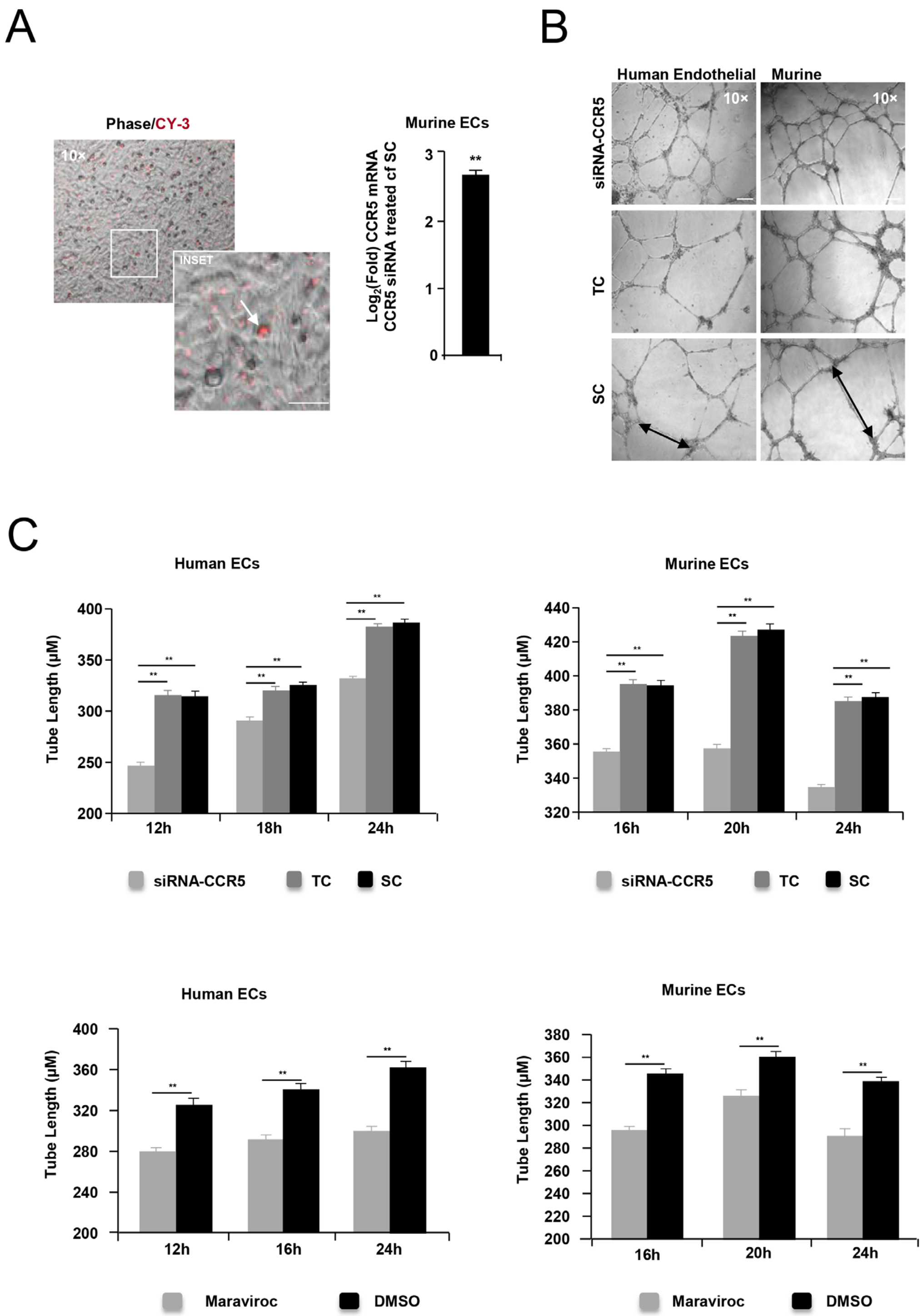

Supplementary Figure S8: siRNA suppression of CCR5 in endothelial cells. A. *Left*, Representative image of endothelial cells (ECs) transfected with Cy3-labeled siGLO RISC-Free Control siRNA (arrow), with inset. Scale bar, 100 µm. *Right*, Q-PCR analysis showing reduction of CCR5 mRNA in murine endothelial cells (MHEVCs) following transfection with siRNA targeting CCR5, compared with scrambled control (SC). Data is represented as mean Log_2(Fold) ± S.E.M. **B.** Representative images of the results of tube formation with human and murine endothelial cells after transfection with CCR5 siRNA, Cy3-labeled siGLO™ RISC-Free scramble (SC) or transfection reagent (TC) controls. Arrows indicate measurement of tube length. Scale bar, 200 µm. **C.** Significant decrease of tube length in human and murine endothelial cells after treatment with either CCR5 siRNA, or maraviroc, compared with controls. Data is represented as mean tube length (µm) ± SEM, and data analyzed by Unpaired *t* test (‘*’$P < 0.05$, ‘**’$P < 0.01$; $\alpha = 0.05$).

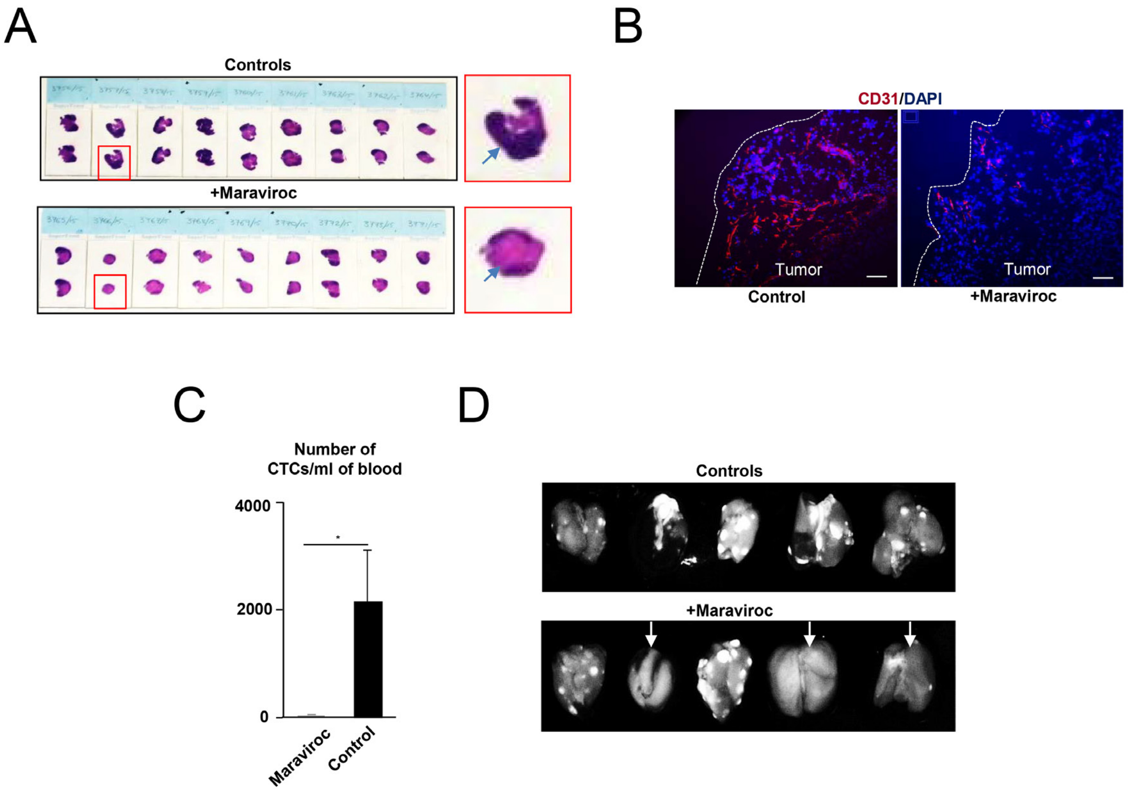

Supplementary Figure S9: Results of maraviroc treatment on tumor growth and vascularization. A. Haematoxylin & eosin (H&E) stained slides showing decrease in growing margin in tumors from maraviroc-treated animals, with inset. **B.** CD31 immunostaining of tumor harvested from maraviroc and control (DMSO) treated animals, showing decreased vascularisation in tumors harvested (Day 13) from maraviroc-treated animals, compared with controls. Resolution 10×, Scale 200 µm. **C.** *Left*, Results of circulating tumor cell (CTC) analysis. 50-100 µm of mouse blood was plates into 10 cm dishes in complete medium and colonies counted after 10 days. *Shown*, significantly fewer viable CTCs in the blood of maraviroc-treated animals, compared with controls (Day 28). Data represented as mean number of CTC colonies ± S.E.M. and analyzed by Unpaired *t* test ('*' $P < 0.05$). **D.** Whole mount of lungs harvested from mice and imaged for mCherry (Day 28). Shown, absence of detectable mCherry^{+} macrometastases in three out of five lungs from maraviroc-treated animals, compared with all the lungs in untreated controls, showing multiple lesions (white arrow).

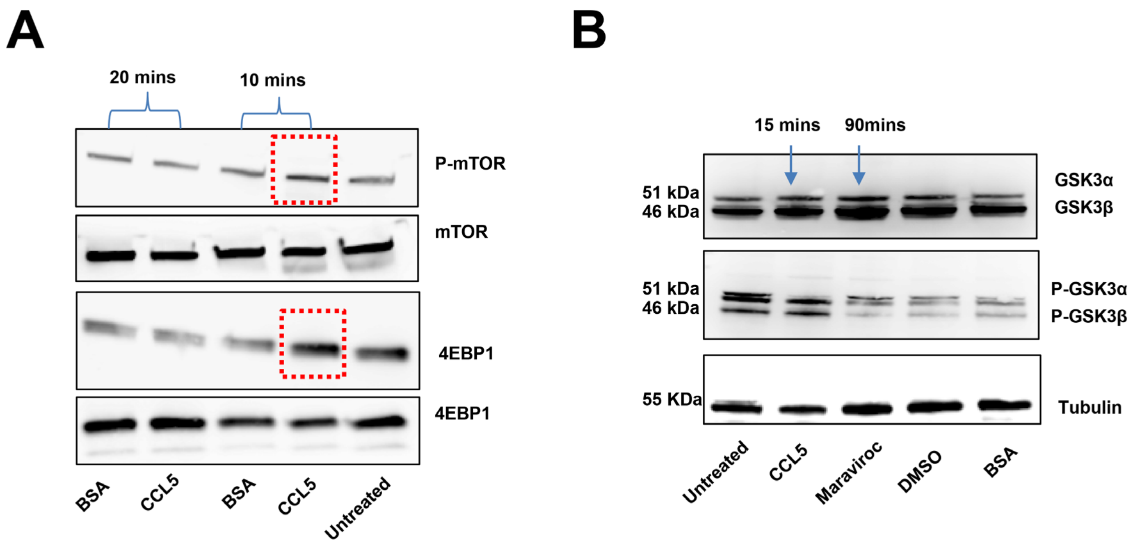

Supplementary Figure S10: Western blot analysis of AKT pathway members in endothelial cells. A. Representative, western blot analysis of lysates obtained from maraviroc, and CCL5 treated endothelial cells (ECs), showing, increase in phosphorylation (P) of key downstream targets of AKT signaling, 4EBP1 (Ser2481) and mTOR (Thr37/46), at 10 and 20 mins. **B.** Western blot analysis of lysates obtained from maraviroc, and CCL5 treated ECs, showing increase in phosphorylation (P) of GSK-3α (Ser21) and GSK-3β (Ser9), with CCL5 treatment (15 min) and decrease in phosphorylation following maraviroc treatment (90 min).

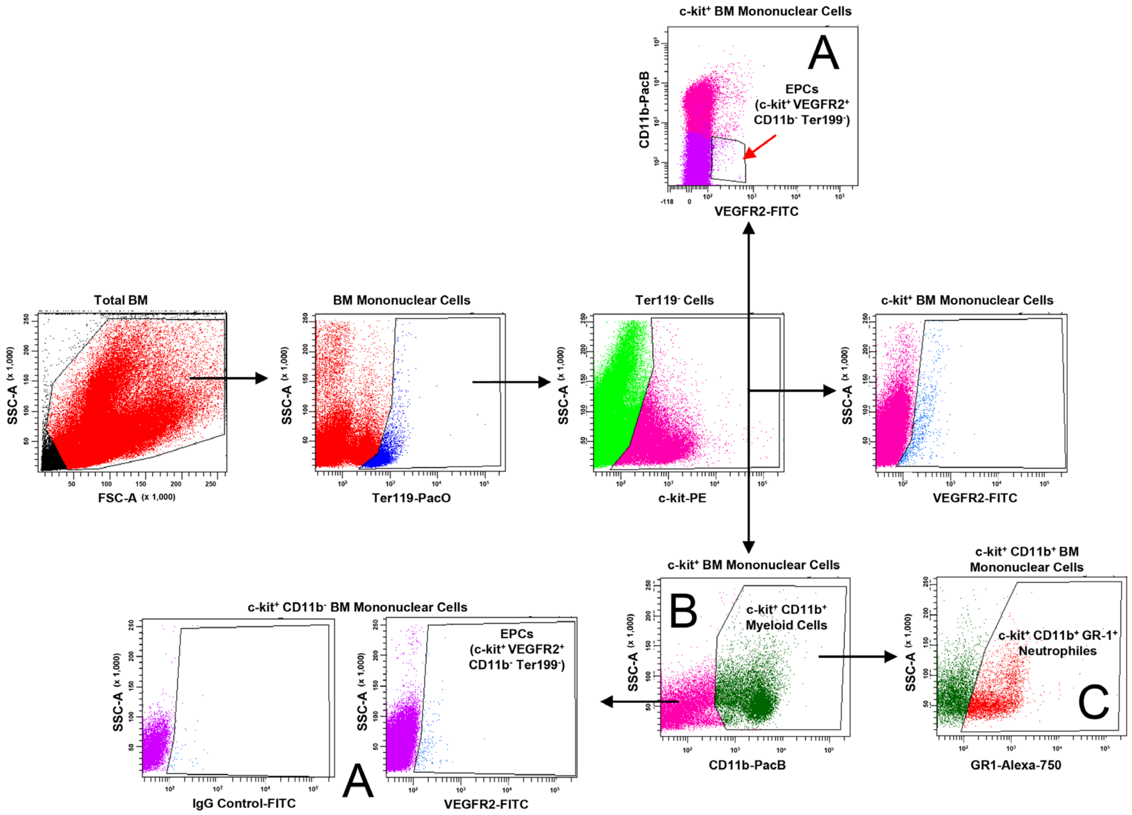

Supplementary Figure S11: Representative scatter plot analysis of total bone marrow (BM) showing identification of Ter119$^-$ c-kit$^+$ CD11b$^-$ VEGFR2$^+$ EPCs and control A. Ter119$^-$ c-kit$^+$ CD11b$^+$ myeloid progenitors (MPs) B. and Ter119$^-$ c-kit$^+$ CD11b$^+$ GR1$^+$ neutrophil progenitors (NPs) C. Analysis was conducted using BD FACS Diva software on the BD Fortessa.

Supplementary Table S1A: Q-PCR short hairpin screening CCL5 mRNA

See Supplementary File 1

Supplementary Table S1B: ELISA:4T1 cells

See Supplementary File 1

Supplementary Table S1C: ELISA:EO771 cells

See Supplementary File 1

Supplementary Table S1D: LV stability measured by mCherry & GFP signal

See Supplementary File 1

Supplementary Table S1E: EO771:EF_{long}-eGFP-Ω cell growth assay

See Supplementary File 1

Supplementary Table S1F: EO771:PGK-mCherry cell growth assay

See Supplementary File 1

Supplementary Table S1G: 4T1:EF_{long}-eGFP-Ω cell growth assay

See Supplementary File 1

Supplementary Table S2A: Tumor growth following CCL5 KD in EO771 cells

See Supplementary File 2

Supplementary Table S2B: Tumor growth following CCL5 KD in 4T1 cells using shRNAi (2)

See Supplementary File 2

Supplementary Table S2C: Tumor growth following CCL5 KD in 4T1 cells using shRNAi (3)

See Supplementary File 2

Supplementary Table S2D: EO771:Ω tumor cell analysis

See Supplementary File 2

Supplementary Table S2E: EO771:Ω PB cell analysis

See Supplementary File 2

Supplementary Table S2F: EO771:Ω BM cell analysis

See Supplementary File 2

Supplementary Table S3A: Tumor growth EO771 $CCR^{-/-}$ KO mice

See Supplementary File 3

Supplementary Table S3B: Vascular branching

See Supplementary File 3

Supplementary Table S3C: % Vascular density

See Supplementary File 3

Supplementary Table S3D: Tumor ECs

See Supplementary File 3

Supplementary Table S3E: BM EPC analysis tumor vs non-tumor

See Supplementary File 3

Supplementary Table S4A: Survival

See Supplementary File 4

Supplementary Table S4B: Lung lesions

See Supplementary File 4

Supplementary Table S5A: Tumor growth in BMT animals

See Supplementary File 5

Supplementary Table S5B: Vascular branching

See Supplementary File 5

Supplementary Table S5C: $CCR5^+$ Tumor ECs

See Supplementary File 5

Supplementary Table S5D: $CCR5^+$ BM-EPCs analysis

See Supplementary File 5

Supplementary Table S5E: BM-EPC analysis

See Supplementary File 5

Supplementary Table S5F: BM-MP analysis

See Supplementary File 5

Supplementary Table S5G: BM-NP analysis

See Supplementary File 5

Supplementary Table S6A: Tumor-conditioned media induction of CCR5 mRNA in mouse and human ECs

See Supplementary File 6

Supplementary Table S6B: CCR5^{+} endothelial cells

See Supplementary File 6

Supplementary Table S6C: CCR5^{+} CD31^{+} vasculature

See Supplementary File 6

Supplementary Table S7A: Murine CCR5 mRNA levels

See Supplementary File 7

Supplementary Table S7B: Tube length murine endothelial cells: CCR5 siRNA

See Supplementary File 7

Supplementary Table S7C: Tube length human endothelial cells: CCR5 siRNA

See Supplementary File 7

Supplementary Table S8A: Tumour Volume 4T1 Cells Maraviroc Treatment

See Supplementary File 8

Supplementary Table S8B: Tumor Weight

See Supplementary File 8

Supplementary Table S8C: Spleen Weight (Day 27)

See Supplementary File 8

Supplementary Table S8D: CTCs

See Supplementary File 8

Supplementary Table S9A: Transwell Assays, CCL5

See Supplementary File 9

Supplementary Table S9B: Transwell Assays, siRNA CCR5

See Supplementary File 9

Supplementary Table S10A: PathScan® Akt Signaling Antibody Array Kit, Chemiluminescent Readout

See Supplementary File 10

Supplementary Table S10B: Quantitative Western Blot Analysis

See Supplementary File 10
