# Supplementary material for: Cancer cell CCL5 mediates bone marrow independent angiogenesis in breast cancer
Source: Oncotarget. 2016 Nov 16;7(51):85437–49. doi: 10.18632/oncotarget.13387 (PMC5356747; doi:10.18632/oncotarget.13387)
Supplement: Supplementary file 2 [file oncotarget-07-85437-s002.doc]

**Table S1A Q-PCR short hairpin s**creening CCL5 mRNA

|  | **Average (∆CT)** | **∆∆CT** | ***P*_value_** | **Fold** |
| --- | --- | --- | --- | --- |
| **NSΩ** | 7.40±0.03  0.13 | **-** | **-** | **-** |
| **CCL5Ω(1)^‡^** | 10.18±0.24 | 1.3 | 0.0001^**^ | 2.46↑ |
| **CCL5Ω(2)** | 8.97±0.0.05 | -1.49 | 0.0007^**^ | 2.81↓ |
| **CCL5Ω(3)** | 8.74±0.12 | -0.27 | 0.0287^*^ | 1.21↓ |
| **CCL5Ω(4)** | 8.78±0.14 | -0.04 | 0.2433 | 1.03↓ |

^*^*P*_value_<0.05, ^**^*P*_value_<0.01, by Unpaired *t* test (α=0.05, one tailed). **^‡^**Seed sequences 1-5, by RNAi codex.

**Table S1**B ELISA:4T1 cells

|  | **Media** | ***P*_value_** | **Lysate** | ***P*_value_** |
| --- | --- | --- | --- | --- |
| **NSΩ** | 238.15±5.92^†^ | - | 179.67±8.67 | - |
| **4T1 WT** | 432.17±27.22 | 0.0011^**^ | 337.04±13.55 | 0.0003^**^ |
| **CCL5Ω(1)** | 123.27±0.86 | <0.0001^**^ | 188.78±3.79 | 0.1950 |
| **CCL5Ω(2)** | 90.65±2.60 | <0.0001^**^ | 115.88±10.28 | 0.0045^**^ |
| **CCL5Ω(3)** | 119.91±1.70 | <0.0001^**^ | 157.58±9.22 | 0.0780 |
| **CCL5Ω(4)** | 147.96±2.12 | <0.0001^**^ | 215.30±10.36 | 0.0289^**^ |

^†^Mean (pg/mg Protein)±S.E.M. *P*_value_<0.01^**^, by Unpaired *t* test (α=0.05, one tailed).

**Table S1**C ELISA:EO771 cells

|  | **Media** | ***P*_value_** | **Lysate** | ***P*_value_** |
| --- | --- | --- | --- | --- |
| **NSΩ** | 146.13±7.91^†^ | **-** | 420.41±5.58 | **-** |
| **EO771 WT** | 81.98±2.96 | 0.0008^**^ | 200.16±3.17 | <0.0001^**^ |
| **CCL5Ω(2)** | 48.40±0.55 | 0.0001^**^ | 116.83±0.98 | <0.0001^**^ |

^†^Mean (pg/mg Protein)±S.E.M. ^**^*P*_value_<0.01, by Unpaired *t* test (α=0.05, one tailed).

**Table S1D LV stability measured by mCherry & GFP s**ignal

| **Hours Post Seeding** | **EO771** | **mCherry signal** | **GFP signal** |
| --- | --- | --- | --- |
| 24h | PGK-mCherry | 87.00^†^±1.00%^‡^ | - |
|  | EF_long_-GFPCCL5Ω | - | 74.00±1.00% |
|  | EF_long_-GFPNSΩ | - | 78.00±3.00% |
| 48h | PGK-mCherry | 94.50±0.50% | - |
|  | EF_long_-GFP-CCL5Ω | - | 76.50±5.50% |
|  | EF_long_-GFP-NSΩ | - | 80.50±1.50% |
| 72h | PGK-mCherry | 96.00±1.00% | - |
|  | EF_long_-GFP-CCL5Ω | - | 75.00±2.00% |
|  | EF_long-_GFP-NSΩ | - | 77.00±2.00% |
| 96h | PGK-mCherry | 95.50±1.50% | - |
|  | EF_long_-GFP-CCL5Ω | - | 73.50±0.50% |
|  | EF_long_-GFP-NSΩ | - | 80.00±1.00% |

^†^RFP/GFP^+^ cells as a % total±S.E.M. Analysis by Unpaired *t* test (α=0.05, one tailed).  ^‡^No significant difference b/n 24 & 96hs.

**Table S1E EO771:EF_long_-eGFP-Ω cell growth assay**

| **Hours post seeding** | **24h** | **48h** | **72h** |
| --- | --- | --- | --- |
| **WT** | 24.96×10^4^±2.76×10^4†^ | 80.055×10^4^±5.805×10^4^ | 153.63×10^4^±9.45×10^4^ |
| **EF_long_-eGFP-NSΩ** | 28.62×10^4^±8.82×10^4^ | 77.085×10^4^±0.95×10^4^ | 154.44×10^4^±13.50×10^4^ |
| **EF_long_-eGFP-CCL5Ω** | 13.26×10^4^±2.64×10^4^ | 81.81×10^4^±6.48×10^4^ | 170.64×10^4^±7.29×10^4^ |

**Table S1F EO771:PGK-mCherry cell growth assay**

| **Hours post seeding** | **24h** | **48h** | **72h** |
| --- | --- | --- | --- |
| **WT** | 14.1×10^4^±0.18×10^4^ | 39.15×10^4^±7.56×10^4^ | 121.84×10^4^±0.01×10^4^ |
| **PGK-mCherry:EF_long_-eGFP-NSΩ** | 12.384×10^4^±2.26×10^4^ | 37.53×10^4^±0.81×10^4^ | 81.27×10^4^±3.78×10^4^ |
| **PGK-mCherry:EF_long_-eGFP-CCL5Ω** | 11.742×10^4^±1.22×10^4^ | 38.88×10^4^±0.54×10^4^ | 101.655×10^4^±2.57×10^4^ |

**Table S1G 4T1:EF_long_-eGFP-Ω cell growth assay**

| **Hours post seeding** | **24h** | **48h** | **72h** |
| --- | --- | --- | --- |
| **WT** | 5.46×10^5^±1.53×10^4^ | 9.62×10^5^±4.69×10^4^ | 2.09×10^6^±8.78×10^4^ |
| **EF_long_-eGFP-NSΩ** | 1.07×10^6^±6.44×10^4^ | 1.21×10^6^±2.25×10^4^ | 2.88×10^6^±2.55×10^5^ |
| **EF_long_-eGFP-CCL5Ω** | 9.64×10^5^±1.27×10^5^ | 1.22×10^6^±6.33×10^4^ | 2.95×10^6^±7.45×10^4^ |

For E-G, ^†^Mean cell number±S.E.M. ^*^*P*_value_<0.05, by MANOVA (α=0.05).
