# Supplementary material for: Cancer cell CCL5 mediates bone marrow independent angiogenesis in breast cancer
Source: Oncotarget. 2016 Nov 16;7(51):85437–49. doi: 10.18632/oncotarget.13387 (PMC5356747; doi:10.18632/oncotarget.13387)
Supplement: Supplementary file 3 [file oncotarget-07-85437-s003.doc]

**Table S2A Tumor growth following CCL5 KD in EO771 cells.**

| **Day** | **EO771:CCL5Ω(2)** | **EO771:NSΩ** |
| --- | --- | --- |
| **6** | 4.46±2.89^†‡^ | 15.33±8.37 |
| **8** | 10.87±4.07 | 27.42±12.91 |
| **10** | 26.59±9.35 | 48.75±24.91 |
| **12** | 39.08±11.53 | 94.16±50.92 |
| **14** | 79.66±21.65 | 149.74±65.17 |
| **16** | 105.46±29.02 | 224.21±116.25 |
| **18** | 180.47±52.10 | 320.78±118.75 |
| **20** | 218.51±56.70 | 539.94±251.04 |
| **22** | 251.96±64.78 | 626.88±230.59 |
| **24** | 438.76±98.78 | 884.93±308.55 |
| **26** | 535.94±92.30 | 1209.51±279.61 |

^†^ Mean volume (mm^3^)±S.E.M. ^**^*P*_value_<0.01, by MANOVA (α=0.05). ^‡^repeat r^2^=0.8733, Pearson’s (*P*_value_<0.0001).

**Table S2B Tumor growth following CCL5 KD in 4T1 cells using shRNAi (2).**

| **Day** | **4T1:WT** | **4T1:CCL5Ω(2)** | **4T1:NSΩ** |
| --- | --- | --- | --- |
| **6** | 11.30±1.95^†‡^ | 9.22±1.47 | 14.21±1.78 |
| **8** | 22.86±2.57 | 29.22±3.16 | 29.58±3.34 |
| **10** | 62.81±7.63 | 38.06±3.76 | 52.15±4.41 |
| **12** | 76.10±7.76 | 30.81±3.42 | 68.29±7.11 |
| **14** | 126.89±13.89 | 31.94±2.91 | 90.91±10.83 |
| **16** | 179.83±17.78 | 37.53±4.31 | 107.21±14.33 |
| **18** | 261.49±31.38 | 52.00±6.56 | 195.82±21.45 |
| **20** | 365.37±47.54 | 75.91±9.79 | 276.64±28.25 |
| **22** | 493.45±61.69 | 135.90±25.11 | 399.78±42.46 |
| **24** | 618.22±81.09 | 210.23±30.04 | 499.85±53.40 |
| **26** | 852.60±112.02 | 328.44±46.56 | 670.04±71.48 |
| **28** | 1073.78±142.80 | 511.40±71.46 | 875.19±90.04 |

^†^Mean volume (mm^3^)±S.E.M. ^**^*P*_value_<0.01, by MANOVA (α=0.05);

^‡^repeat r^2^=0.8733, Pearson’s (*P*<0.0001)

**Table S2C Tumor growth following CCL5 KD in 4T1 cells using shRNAi (3)**

| **Day** | **4T1:CCL5Ω (3)** | **4T1:NSΩ** |
| --- | --- | --- |
| **6** | 5.74±2.61^†‡^ | 6.71±2.01 |
| **8** | 9.92±4.58 | 13.86±4.90 |
| **10** | 15.75±7.53 | 21.49±7.58 |
| **12** | 24.73±11.61 | 32.84±10.36 |
| **14** | 35.23±16.36 | 44.95±11.40 |
| **16** | 52.71±23.09 | 68.06±13.84 |
| **18** | 73.41±30.82 | 101.29±16.45 |
| **20** | 114.89±42.38 | 187.61±37.60 |
| **22** | 175.49±53.38 | 299.72±60.72 |

^†^Mean volume (mm^3^)±S.E.M. ^**^*P*_value_<0.01, by MANOVA (α=0.05).

^‡^repeat r^2^=0.8733, Pearson’s (*P*_value_<0.0001)

**Table S2D EO771:Ω tumor cell analysis**

|  | **EO771:NSΩ** | **EO771:CCL5Ω** | ***P*_value_** | **EO771** | ***P*_value_** |
| --- | --- | --- | --- | --- | --- |
| **Endothelial Cells** | 21.43±2.59%^†^ | 13.74±1.92% | 0.0285^*^ | 19.49±3.22% | 0.3253 |
| **EPCs** | 3.12±0.44% | 1.83±0.23% | 0.0239^*^ | 2.13±0.48% | 0.0851 |
| **MPs** | 29.81±3.83% | 28.22±5.58% | 0.4078 | 22.75±4.16% | 0.1237 |
| **NPs** | 19.57±3.16% | 18.30±5.19% | 0.4165 | 12.16±5.44% | 0.0586 |

^†^Mean % total cells±S.E.M. ^*^*P*_value_<0.05, by Unpaired *t* test (α=0.05, one tailed).

**Table S2E EO771:Ω PB cell analysis**

|  | **EO771:NSΩ** | **EO771:CCL5Ω** | ***P*_value_** | **EO771** | ***P*_value_** | **No Tumor** | ***P*_value_** |
| --- | --- | --- | --- | --- | --- | --- | --- |
| **CEPs** | 3.07±1.18%**^†^** | 0.60±0.147%**^†^** | 0.0330^*^ | 3.15±1.66%**^†^** | 0.4853 | 0.83±0.16%**^†^** | 0.2491 |
| **MPs** | 0.31±0.05%**^‡^** | 0.59±0.12%**^‡^** | 0.0337^*^ | 0.47±0.07%**^‡^** | 0.0442^*^ | 0.43±0.05%**^‡^** | 0.1522 |
| **NPs** | 0.09±0.01%**^‡^** | 0.22±0.04%**^‡^** | 0.0113^*^ | 0.13±0.03%**^‡^** | 0.1932 | 0.20±0.01%**^‡^** | 0.0032^**^ |
| **c-kit^+^** | 0.55±0.08%**^‡^** | 1.49±0.48%**^‡^** | 0.0399^*^ | 0.86±0.10%**^‡^** | 0.0156^*^ | 0.68±0.08%**^‡^** | 0.2118 |

^†^ Mean % of c-kit^+^ cells±S.E.M. ^‡^Mean % PBMNCs±S.E.M. ^*^*P*_value_<0.05, ^**^*P*_value_<0.01, by Unpaired *t* test (α=0.05, one tailed).

**Table S2F EO771:Ω BM cell analysis**

|  | **EO771:NSΩ** | **EO771:CCL5Ω** | ***P*_value_** | **EO771** | ***P*_value_** | **No Tumor** |  | ***P*_value_** |
| --- | --- | --- | --- | --- | --- | --- | --- | --- |
| **EPCs** | 0.34±0.06%**^†^** | 0.36±0.07%**^†^** | 0.4303 | 0.42±0.04%**^†^** | 0.1515 | 0.45±0.04%**^†^** |  | 0.1902 |
| **MPs** | 2.97±0.18%**^‡^** | 2.65±0.082%**^‡^** | 0.0645 | 2.36±0.12%**^‡^** | 0.0079^**^ | 2.02±0.23%**^‡^** |  | 0.0216^*^ |
| **NPs** | 1.25±0.09%**^‡^** | 1.22±0.06%**^‡^** | 0.3653 | 1.03±0.06%**^‡^** | 0.0303^*^ | 0.88±0.13%**^‡^** |  | 0.0488^*^ |
| **c-kit^+^** | 6.23±0.38%**^‡^** | 5.41±0.20%**^‡^** | 0.0417^*^ | 4.88±0.13%**^‡^** | 0.0034^**^ | 4.79±0.32%**^‡^** |  | 0.0491^*^ |

^†^Mean % of c-kit^+^ cells±S.E.M. ^‡^Mean % BMMNCs±S.E.M. ^*^*P*_value_<0.05, ^**^*P*_value_<0.01, by Unpaired *t* test (α=0.05, one tailed).
