# Supplementary material for: Cancer cell CCL5 mediates bone marrow independent angiogenesis in breast cancer
Source: Oncotarget. 2016 Nov 16;7(51):85437–49. doi: 10.18632/oncotarget.13387 (PMC5356747; doi:10.18632/oncotarget.13387)
Supplement: Supplementary file 4 [file oncotarget-07-85437-s004.doc]

**Table S3A Tumor growth EO771 CCR^-/-^** KO mice

| **Day** | **WT** | **CCR1^-/-^** | **CCR5^-/-^** |
| --- | --- | --- | --- |
| **6** | 10.36±3.04^†^ | 11.51±3.02 | 12.69±3.51 |
| **8** | 16.15±4.5 | 17.96±5.59 | 19.12±5.32 |
| **10** | 31.3±7.01 | 41.67±11.10 | 36.37±2.98 |
| **12** | 46.54±10.68 | 73.91±13.69 | 39.65±6.32 |
| **14** | 61.77±14.34 | 106.16±16.27 | 42.93±9.66 |
| **16** | 111.18±20.22 | 137.26±16.07 | 48.05±17.94 |
| **18** | 160.60±26.10 | 168.35±15.87 | 53.17±26.22 |
| **20** | 273.06±63.87 | 261.42±17.55 | 51.94±25.53 |
| **22** | 416.89±83.37 | 368.38±29.08 | 64.84±36.51 |
| **24** | 637.64±138.07 | 553.11±45.45 | 65.90±38.24 |
| **26** | 784.98±101.82 | 1019.91±97.73 | 67.60±40.05 |

^†^ Mean volume (mm)±S.E.M. ^**^*P*_value_<0.01, by MANOVA (α=0.05)

**Table S3B Vascular branching**

| **Day** | **WT** | **CCR1^-/-^** | ***P*_value_** | **CCR5^-/-^** | ***P*_value_** |
| --- | --- | --- | --- | --- | --- |
| **14** | 86.0±12.09^†^ | **-** | **-** | 21.29±5.86 | <0.0001^**^ |
| **28** | 109.5±14.50 | 70.6±7.52 | 0.0006^**^ | 28.26±2.66 | <0.0001^**^ |

^†^Mean branch point/field±S.E.M. ^**^*P*_value_<0.01, by Unpaired *t* test (α=0.05, one tailed)

**Table S3C % Vascular density**

| **Day** | **WT** | **CCR1^-/-^** | ***P*_value_** | **CCR5^-/-^** | ***P*_value_** |
| --- | --- | --- | --- | --- | --- |
| **14** | 7.07±1.12%^†^ | **-** | **-** | 5.51±0.56% | 0.0273^*^ |
| **28** | 6.73±0.36% | 7.67±0.79% | 0.1095 | 7.57±0.71% | 0.1823 |

^†^Mean CD31^+^ % vascular area corrected for tumor area±S.E.M. ^*^*P*_value_<0.05, by Unpaired *t* test (α=0.05, one tailed)

**Table S3D Tumor ECs**

| **Day** | **WT** | **CCR1^-/-^** | ***P*_value_** | **CCR5^-/-^** | ***P*_value_** |
| --- | --- | --- | --- | --- | --- |
| **14** | 0.3224±0.0378%^†^ | 0.0430±0.0092% | 0.0008^**^ | 0.0525±0.0083% | 0.0002^**^ |
| **28** | 0.0079±0.0022% | 0.0076±0.0022% | 0.4690 | 0.0215%±0.0001% | 0.0014^**^ |

^†^Mean % of total tumor cells±S.E.M. ^**^*P*_value_<0.01, by Unpaired *t* test (α=0.05, one tailed).

**Table S3E** BM EPC analysis tumor vs non-tumor

| **Day** | **WT** | **+Tumor** | ***P*_value_** | **CCR5^-/-^** | **+Tumor** | ***P*_value_** |
| --- | --- | --- | --- | --- | --- | --- |
| **14** | 0.08±0.01%^†^ | 0.15±0.01% | 0.0125^*^ | 0.09±0.01% | 0.11±0.01% | 0.0478^*^ |
| **28** | 0.16±0.05% | 0.09±0.01% | 0.1072 | 0.39±0.07% | 0.51±0.06% | 0.0773 |

^†^Mean % of total BMMNC±S.E.M. ^*^*P*_value_<0.05, by Unpaired *t* test (α=0.05, one tailed).
