# Supplementary material for: Cancer cell CCL5 mediates bone marrow independent angiogenesis in breast cancer
Source: Oncotarget. 2016 Nov 16;7(51):85437–49. doi: 10.18632/oncotarget.13387 (PMC5356747; doi:10.18632/oncotarget.13387)
Supplement: Supplementary file 5 [file oncotarget-07-85437-s005.doc]

**Table S4A** Survival

| **Variable** | **Mean** | | **Median** | | | |
| --- | --- | --- | --- | --- | --- | --- |
|  | **Estimate** | **S.E.M.** | **95% Confidence** | | **Estimate** | **Std. Error** |
|  |  |  | **Lower Bound** | **Upper Bound Bound** |  |  |
| **WT** | 26.875 | 2.310 | 22.347 | 31.403 | 27.000 | 4.950 |
| **CCR5^-/-^** | 40.667 | 5.435 | 30.014 | 51.319 | 37.000 | 6.736 |
| **Overall** | 32.786 | 3.206 | 26.502 | 39.069 | 28.000 | 3.742 |

|  | **Chi-Square** | **df** | **Sig.** |
| --- | --- | --- | --- |
| **Log Rank (Mantel-Cox)** | 5.234 | 1 | 0.022^*^ |

^*^*P*_value_<0.05 Comparison of median survival by Chi-square 5.234. 95% confidence.

**Table S4B Lung l**esions

|  | **WT** | **CCR1^-/-^** | ***P*_value_** | **CCR5^-/-^** | ***P*_value_** |
| --- | --- | --- | --- | --- | --- |
| **Haematological** | 10.00±2.46 | 25.00±6.00 | 0.0094^**^ | 12.00±4.02 | 0.3359 |
| **Non-haematological** | 7.36±1.19 | 22.00±3.00 | <0.0001^**^ | 14.67±2.49 | 0.0037^**^ |

Mean number of metastases/lung±S.E.M. ^*^*P*_value_<0.05, ^**^*P*_value_<0.01, by Unpaired *t* test (α=0.05, one tailed).
